# Effects of a liquefied petroleum gas stove intervention on stillbirth, congenital anomalies and neonatal mortality: A multi-country household air pollution intervention network trial^☆^

**DOI:** 10.1016/j.envpol.2024.123414

**Published:** 2024-01-27

**Authors:** Ashley Younger, Wenlu Ye, Abbey Alkon, Kristen Harknett, Miles A. Kirby, Lisa Elon, Amy E. Lovvorn, Jiantong Wang, Anaité Diaz-Artiga, John P. McCracken, Adly Castañaza Gonzalez, Libny Monroy Alarcon, Alexie Mukeshimana, Ghislaine Rosa, Marilu Chiang, Kalpana Balakrishnan, Sarada S. Garg, Ajay Pillarisetti, Ricardo Piedrahita, Michael A. Johnson, Rachel Craik, Aris T. Papageorghiou, Ashley Toenjes, Kendra N. Williams, Lindsay J. Underhill, Stella M. Hartinger, Laura Nicolaou, Howard H. Chang, Luke P. Naeher, Joshua Rosenthal, William Checkley, Jennifer L. Peel, Thomas F. Clasen, Lisa M. Thompson

**Affiliations:** a School of Nursing, University of California, San Francisco, CA, USA; b Division of Environmental Health Sciences, School of Public Health, University of California, Berkeley, CA, USA; c Department of Global Health and Population, Harvard T. H. Chan School of Public Health, Harvard University, Boston, MA, USA; d Department of Biostatistics and Bioinformatics, Rollins School of Public Health, Emory University, Atlanta, GA, USA; e Gangarosa Department of Environmental Health, Rollins School of Public Health, Emory University, Atlanta, CA, USA; f Center for Health Studies, Universidad del Valle de Guatemala, Guatemala City, Guatemala; g Department of Environmental Health Science, College of Public Health, University of Georgia, Athens, GA, USA; h Eagle Research Centre, Kigali, Rwanda; i Department of Disease Control, London School of Hygiene and Tropical Medicine, London, UK; j Department of Environmental Health Engineering, ICMR Center for Advanced Research on Air Quality, Climate and Health, Sri Ramachandra Institute for Higher Education and Research (Deemed University), Chennai, India; k Berkeley Air Monitoring Group, Berkeley, CA, USA; l Nuffield Department of Women’s and Reproductive Health, University of Oxford, Oxford, UK; m Cardiovascular Division, Department of Medicine, Washington University in St. Louis, St. Louis, MO, USA; n Latin American Center of Excellence on Climate Change and Health, Universidad Peruana Cayetano Heredia, Lima, Peru; o Division of Pulmonary and Critical Care, Johns Hopkins University School of Medicine, Baltimore, MD, USA; p Center for Global Non-Communicable Disease Research and Training, School of Medicine, Johns Hopkins University, Baltimore, MD, USA; q Division of Epidemiology and Population Studies, Fogarty International Center, National Institutes of Health, Bethesda, MD, USA; r Department of Environmental and Radiological Health Sciences, Colorado State University, Fort Collins, CO, USA; s Nell Hodgson Woodruff School of Nursing, Emory University, Atlanta, GA, USA

**Keywords:** Cooking fuel, Birth outcomes, Low- and middle-income countries, Congenital anomaly, Stillbirth, Neonatal mortality

## Abstract

Household air pollution (HAP) from cooking with solid fuels used during pregnancy has been associated with adverse pregnancy outcomes. The Household Air Pollution Intervention Network (HAPIN) trial was a randomized controlled trial that assessed the impact of a liquefied petroleum gas (LPG) stove and fuel intervention on health in Guatemala, India, Peru, and Rwanda. Here we investigated the effects of the LPG stove and fuel intervention on stillbirth, congenital anomalies and neonatal mortality and characterized exposure-response relationships between personal exposures to fine particulate matter (PM_2.5_), black carbon (BC) and carbon monoxide (CO) and these outcomes. Pregnant women (18 to <35 years of age; gestation confirmed by ultrasound at 9 to <20 weeks) were randomly assigned to intervention or control arms. We monitored these fetal and neonatal outcomes and personal exposure to PM_2.5_, BC and CO three times during pregnancy, we conducted intention-to-treat (ITT) and exposure-response (E-R) analyses to determine if the HAPIN intervention and corresponding HAP exposure was associated with the risk of fetal/neonatal outcomes. A total of 3200 women (mean age 25.4 ± 4.4 years, mean gestational age at randomization 15.4 ± 3.1 weeks) were included in this analysis. Relative risks for stillbirth, congenital anomaly and neonatal mortality were 0.99 (0.60, 1.66), 0.92 (95 % CI 0.52, 1.61), and 0.99 (0.54, 1.85), respectively, among women in the intervention arm compared to controls in an ITT analysis. Higher mean personal exposures to PM_2.5_, CO and BC during pregnancy were associated with a higher, but statistically non-significant, incidence of adverse outcomes. The LPG stove and fuel intervention did not reduce the risk of these outcomes nor did we find evidence supporting an association between personal exposures to HAP and stillbirth, congenital anomalies and neonatal mortality.

## 1. Introduction

Globally, 3.8 billion people are exposed to household air pollution (HAP) from the burning of solid fuels (e.g, wood, charcoal, dung, agricultural residue) for cooking and heating (Health Effects Institute, 2020; Ghosh et al., 2021). In 2019, HAP exposure contributed to approximately 2.3 million deaths, the majority of which occurred in South Asia (36 %), sub-Saharan Africa (30 %), and Southeast Asia, East Asia and Oceania (28 %) (Health Effects Institute, 2020). In an effort to protect population health, the World Health Organization (WHO) revised the Global Air Quality Guidelines (AQGs) for annual mean fine particulate matter (PM_2.5_, with a diameter of 2.5 μm or less) exposure from 10 μg/m^3^ in 2005 to 5 μg/m^3^ in 2021 (WHO, 2021). To date, the relationship between HAP exposure and fetal and neonatal outcomes has been inconclusive, though indicative of potential effects.

The incomplete combustion of unclean cooking fuels, which includes both solid fuels and liquid kerosene, releases particulate matter (PM), black carbon (BC), carbon monoxide (CO), and other pollutants such as nitrogen dioxide (NO_2_) and sulfur dioxide (SO_2_) that adversely impact health. Because particulate matter can cross the placenta and lead to pathological changes, including chronic placental hypoxia and thrombotic lesions, exposure to HAP during pregnancy can negatively affect fetal growth and development (Dutta et al., 2018; Kannan et al., 2006; Wylie et al., 2017). Three systematic reviews and meta-analyses reported associations between exposure to unclean cooking fuel during pregnancy and adverse birth outcomes including reductions in birth weight, increased risk of low birthweight, and stillbirth (Amegah et al., 2014; Lee et al., 2020; Pope et al., 2010).

The WHO estimates 295,000 newborns die each year due to congenital anomalies; 95 % of these deaths occur in low- and middle-income countries (LMICs) (WHO, 2023). It has been hypothesized that maternal exposure to environmental risk factors, including air pollutants, may contribute to the incidence of abnormal fetal development by promoting oxidative stress (Kampa et al., 2008). Prenatal exposure to HAP was a risk factor for cleft lip and/or palate in children in a population-sampled case-control study across 7 low-resource countries (Auslander et al., 2020). A recent systematic review and meta-analysis by Ravindra et al. (2021) reported that prenatal exposure to ambient PM_2.5_ and NO_2_ significantly increased the prevalence of pulmonary valve stenosis, PM_2.5_ with tetralogy of Fallot, SO_2_ with ventral septal defect and cleft lip/cleft palate, and O_3_ with increased prevalence of limb defects (Ravindra et al., 2021). Most studies included in the review relied on ambient air pollutant concentrations measured at stationary monitoring stations and lacked measures of indoor or personal measures of air pollution exposure.

The United Nations Inter-agency Group for Child Mortality Estimation (UN IGME) estimated 2 million stillbirths occur annually with 84 % occurring in LMICs (Hug et al., 2020). In a systematic review and meta-analysis, Amegah et al. (2014) reported a 29 % increased risk of stillbirth from solid fuel use, based on 5 observational studies (summary-effect estimates [EE] 1.29, 95 % CI: 1.18, 1.41) (Amegah et al., 2014). Alexander et al. (2018) conducted the first randomized controlled trial (RCT) evaluating the impact of an ethanol stove intervention versus continued use of kerosene/firewood on stillbirth. The study reported an overall small number of stillbirths (n = 10) and no statistically significant difference between the intervention and control arms (EE 0.6, 95 % CI: 0.2, 1.9) (Alexander et al., 2018).

According to estimates from the WHO, 2.4 million infants died in the first month of life in 2019 (WHO, 2022). A prospective cohort study in rural India, Pakistan, Kenya, Zambia and Guatemala reported households using polluting fuels increased the risk of very early neonatal mortality (adjusted Odds Ratio [aOR] 1.82, 95 %CI: 1.47, 2.22) (Patel et al., 2015). In contrast, a study using the Bangladesh Demographic Health Survey from 2004 to 2014 did not find an effect from exposure to polluting cooking fuel on increased odds of neonatal mortality compared to clean fuels such as electricity, LPG and gas (aOR 1.25, 95 % CI: 0.85, 1.84) (Nisha et al., 2018). However, an RCT in urban Nigeria did not detect a statistically significant difference in neonatal mortality between the intervention (ethanol stove) and control arms (EE 0.4, 95 % CI: 0.1, 1.4) (Alexander et al., 2018).

Prior research examining the association between unclean fuel use during pregnancy and these fetal and neonatal outcomes is primarily cross-sectional (Younger et al., 2022). Three recent trials in Nepal, Ghana and Nigeria have yielded mixed results, making it difficult to conclude that there are significant associations between cleaner fuel interventions and birth outcomes (Alexander et al., 2018; Katz et al., 2016; Quinn et al., 2021a). The Household Air Pollution Intervention Network (HAPIN) randomized controlled trial of LPG stoves and continuous, free fuel distribution with behavioral reinforcement to intervention adherence occurred in four diverse LMICs. The primary outcomes of the trial were birthweight, stunting, severe pneumonia in children under 1 year of age and systolic blood pressure in older adult women. The fetal and neonatal outcomes presented here were exploratory outcomes of the main HAPIN Trial. The objectives of this paper are to 1) investigate if adverse fetal and neonatal outcomes (stillbirth, congenital anomalies and neonatal mortality) differ based on stove type across the four research sites within the HAPIN Trial, and 2) characterize the exposure-response relationships between personal exposure to PM_2.5_, BC, and CO, and fetal and neonatal outcomes of interest.

## 2. Materials and methods

### 2.1. Trial design and study settings

The HAPIN trial was conducted in 3200 households from four intervention research centers (IRCs) in Guatemala, Rwanda, India (2 study sites), and Peru (6 study sites) (Clasen et al., 2020). Participating women were followed from enrollment during pregnancy through the first year of the infant’s life. Fidelity and adherence to the intervention were evaluated using stove and fuel delivery records, questionnaires, visual observations, and temperature-logging stove use monitors (SUMs) that continuously monitored traditional stoves in intervention homes throughout the trial (Johnson et al., 2020; Wilson et al., 2020). Biomarker data on pregnant women were also collected as part of the HAPIN study design and are discussed elsewhere (Barr et al., 2020). Among participants in the LPG intervention arm, 96 % reported cooking exclusively with LPG at the two follow-up visits during the prenatal period. Among those who retained the traditional stove (68.6 %), the majority (59.5 %) did not use them (Quinn et al., 2021b). Information detailing behavior change strategies to promote exclusive LPG use in the HAPIN trial have been published (Williams et al., 2020).

The study protocol has been reviewed and approved by institutional review boards (IRBs) or Ethics Committees at Emory University (00089799), Johns Hopkins University (00007403), Sri Ramachandra Institute of Higher Education and Research (IEC-N1/16/JUL/54/49) and the Indian Council of Medical Research – Health Ministry Screening Committee (5/8/4–30/(Env)/Indo-US/2016-NCD-I), Universidad del Valle de Guatemala (146-08-2016) and Guatemalan Ministry of Health National Ethics Committee (11–2016), Asociación Beneficia PRISMA (CE2981.17), the London School of Hygiene and Tropical Medicine (11664-5) and the Rwandan National Ethics Committee (No.357/RNEC/2018), and Washington University in St. Louis (201611159). The study has been registered with ClinicalTrials.gov (Identifier NCT02944682).

### 2.2. Participant recruitment and enrollment

In cooperation with local ministries of health, eligible pregnant women were identified at antenatal clinics. At each research site, 800 pregnant women (aged 18 to <35 years, 9 to <20 weeks gestation confirmed by ultrasound) who primarily used traditional solid fuel stoves for cooking were recruited (Clasen et al., 2020). Trained study personnel conducted ultrasounds at antenatal clinics using a portable Edge ultrasound (Fujifilm-SonoSite Inc., Bothell, WA, USA). Eligibility was based on having a viable (detectable heart rate on ultrasound), singleton pregnancy between 9 weeks 0 days and 19 weeks 6 days of gestation confirmed by ultrasound (Dávila-Romá et al., 2021). Informed consent was obtained from all study participants that met eligibility requirements using standard procedures. Participants were able to withdraw from the study at any time.

### 2.3. Randomization and intervention

Half of the participating households in each site were randomly assigned to receive an LPG cookstove and free fuel delivered to their home (intervention arm). Education and behavioral reinforcements occurred in intervention homes that continued to use traditional stoves. The other half (control arm) were anticipated to continue cooking with solid fuels. The Emory University data management core assembled randomization lists and sent the assignments to the four participating IRCs in sealed tamper-proof envelopes. The randomization list was further stratified into two sites in India and six sites in Peru. Trained field staff visited the homes of eligible participants and randomized participants into intervention and control after the participants selected one of six envelopes provided to them. The intervention households received a high-quality, locally available LPG stove and a continuous supply of free LPG fuel for the duration of the pregnancy and through the child’s first year of life. Control households received specific compensations approved by local IRBs or ethics committees during or after the study (Quinn et al., 2019).

### 2.4. Measurement of exposures, outcomes, and covariates

A baseline survey was administered by a trained, local field staff or nurse following recruitment and informed consent. The baseline survey included questions about cooking behaviors, household characteristics, socioeconomic and demographic information, medical and obstetric history, physical activity, dietary diversity using the FAO Minimum Diet Diversity for Women (MDD-W), and household food insecurity using the FAO Food Insecurity Experience Scale (FAO & FHI 360, 2016; Ballard et al., 2013). Field staff also measured resting blood pressure in triplicate, and weight and height in duplicate of the enrolled pregnant women at this visit (Clasen et al., 2020). Additional home visits occurred two additional times before birth, at 24–28 weeks and 32–36 weeks gestation. At these visits, field staff repeated many of the same procedures that occurred at the baseline visit.

We measured 24-hr personal exposure to PM_2.5_, BC, and CO three times during pregnancy: once at baseline and twice post-randomization, along with household surveys and health assessments. Personal PM_2.5_ exposure was measured using the Enhanced Children’s MicroPEM^™^ (ECM, RTI International, Research Triangle Park, NC). The ECM is a small, lightweight, and quiet PM_2.5_ nephelometric and gravimetric sampler (Johnson et al., 2020) that collects PM_2.5_ mass on a 15 mm Teflon^®^ filters (PT15-AN-PF02; MTL Corporation). BC exposure was quantified on sampled PM_2.5_ filters using a SootScan^™^ Model OT21 transmissometer (Magee Scientific, Berkeley, CA) (Johnson et al., 2020). Real-time personal exposure to CO was measured with Lascar CO monitors (model EL-USB-300, Lascar Electronics, Erie, PA). Participants wore samplers on a shoulder band or in the pocket of a customized garment near their breathing zone. Exposure assessment procedures and data processing have been published elsewhere (Johnson et al., 2020).

Health outcome data were extracted from Adverse Event (AEs) and Severe Adverse Event (SAEs) case report forms, study exit forms, and verbal autopsies that were conducted within 30 days of the infant death. Outcomes were defined according to standard definitions used in clinical trials (OHRP, 2007). Whenever an event occurred, the field staff collected detailed information on the appropriate case report form (AE or SAE) in REDCap^™^ during site visits. If any health condition was ongoing and required medical attention, a referral to the nearest health center or hospital was made. We define *stillbirth* as a fetal death ≥20 weeks gestation based on dates established at the baseline (ultrasound or last menstrual period), *neonatal mortality* as the death of any live-born infant in first 28 completed days of life, and *congenital anomaly* as any structural or functional anomalies that occur during intrauterine life.

### 2.5. Statistical analysis

We used multivariable logistic regression to characterize the exposure-response relationship between personal PM_2.5_/BC/CO exposures and stillbirth, congenital anomalies and neonatal mortality outcomes, controlling for confounders. The general model specification is as follows:

(1) $$logit(Y_{i})=\beta_{0}+\beta_{1}(Exposure_{i})+\sum \beta Z_{i}$$

where $Y_{i}$ is the expected occurrence of the outcome of subject i, $\beta_{0}$ is the population intercept, $\beta_{1}$ is the exposure coefficient of interest, $Exposure_{i}$ is the weighted mean PM_2.5_/BC/CO exposure over gestation, and $Z_{i}$ are time-independent covariates (i.e., IRC, maternal age, nulliparity, mother’s highest education level, baseline BMI, baseline hemoglobin level, exposure to secondhand smoke at home, household food insecurity, and mother’s diet diversity scores).

For the control group, the weighted mean personal exposure was simply the mean of all available measurements. For the intervention group, we weighted the baseline exposure and the mean of post-baseline exposures, with the weight for the baseline exposure being the gestational age before intervention, and the weight for the mean post-baseline exposures being the duration of gestation with the intervention. We used a weighted mean exposure to give more weight to the baseline measurement for participants in the intervention group when the intervention occurred later (Balakrishnan et al., 2023; Ye et al., 2022).

Covariates included in the models were based on previous literature and data availability. Results are expressed as the odds ratios with 95 % confidence interval (CI) of outcome occurrence per unit increase in PM_2.5_/BC/CO exposures. We also assessed effect modification by BMI, maternal age, and gestational age at baseline for the primary analyses. As secondary analyses, we investigated associations between the outcomes and the mean of post-intervention exposures. All analyses were conducted using R version 4.0.3 (Comprehensive R Archive Network: http://cran.r-project.org).

We present exploratory outcomes for the HAPIN trial, for which there were no sample size calculations. Statistical significance was set *a priori* at the 0.05 level. The statistical analyses were performed in accordance with a pre-specified plan, and data analysts were initially blinded to study arm assignment. First, we conducted preliminary descriptive analyses of baseline data summarized by frequencies and percentages for categorical variables and by means and standard deviation (SD) for continuous variables; missing data are reported separately. Second, outcomes were compared using two sample t-tests for continuous variables and chi-square tests for categorical variables. Third, we used intention-to-treat (ITT) analyses according to the randomized allocation. Binary outcomes of stillbirth, neonatal death, and congenital anomalies were compared between the intervention and control arms using log binomial regression models. Model equations are generalized as follows:

(2) $$\log(Y_{i})=\beta_{0}+\beta_{1}Arm_{i}+\beta_{2}X_{1i}+\cdots +\beta_{11}X_{10i}$$

For all binary outcomes $Y_{i}$, we performed two-tailed hypothesis tests at an ***α***-level of 0.05, and calculated risk ratios. Since adverse outcome rates were expected to be low, we created a composite score by summing stillbirths, congenital anomalies and neonatal deaths into one binary (yes/no) adverse neonatal and fetal outcome. The composite score accounted for multiple outcomes in the same participant (example: congenital anomaly and stillbirth in same participant counted as one event). $Arm_{i}$ is an indicator variable (0 for control and 1 for intervention) for participant i. We also controlled for 10 randomization strata ($X_{1i}$ through $X_{10i}$) in the ITT models.

## 3. Results

### 3.1. Baseline characteristics

**Fig. 1.**
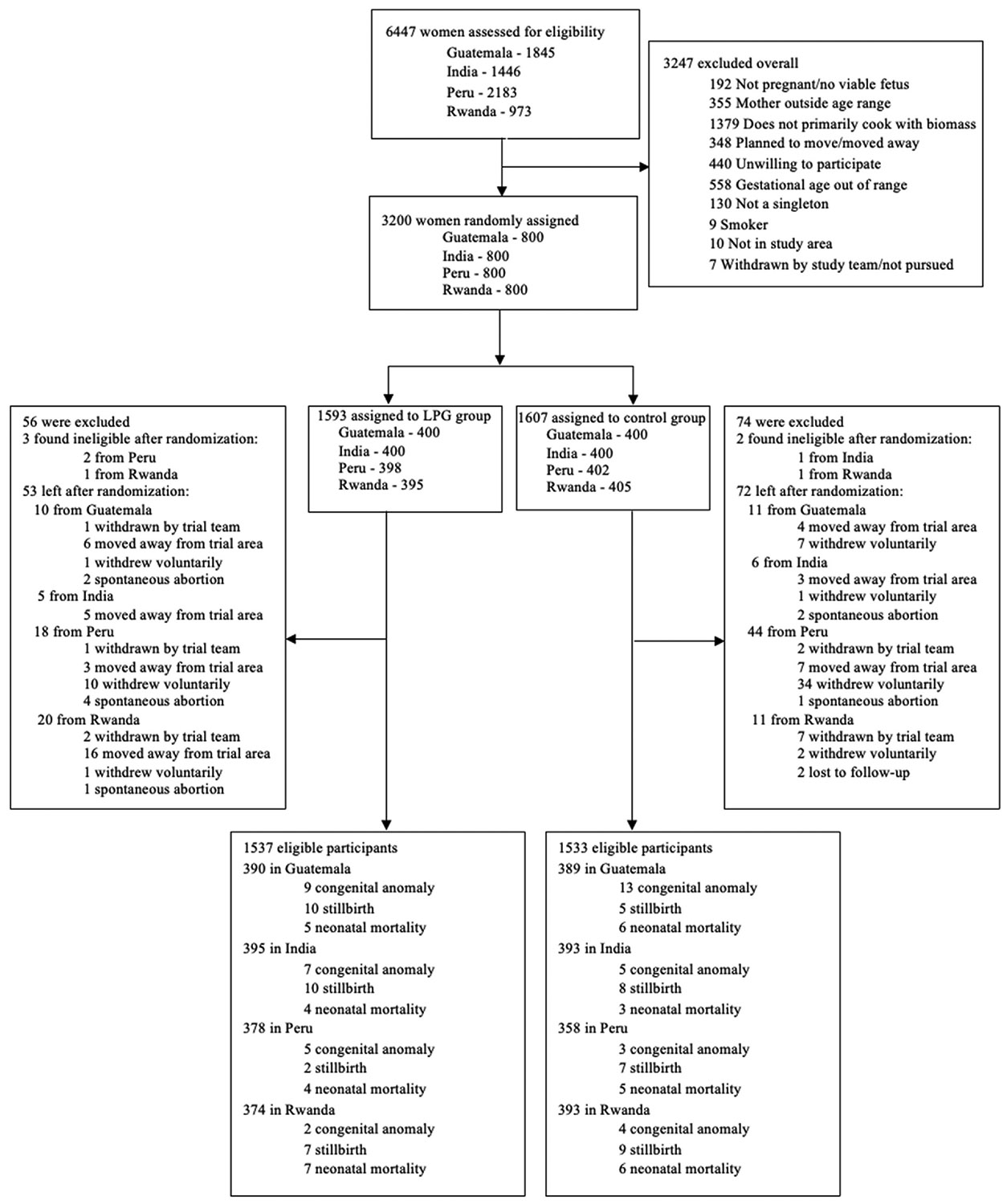
CONSORT flow chart.

Baseline characteristics are described by intervention versus control arms in Table 1. After 5 households were determined to be ineligible after randomization and exited the study, a total of 3195 pregnant women were randomized to the intervention (1590) or control arm (1605) as seen in the Consolidated Standards of Reporting Trials (CONSORT) flow diagram presented in Fig. 1 (Moher et al., 2010). There were no significant differences in baseline characteristics between study arms. The mean (standard deviation [SD]) gestational age of enrollment was 15.4 (3.1) weeks overall and was similar across the 4 IRCs, indicating the majority of the women were enrolled early in the second trimester. Nearly thirteen percent of pregnant participants were less than 20 years old; most were 20–24 (37.4 %) and 25–29 (31.8 %). Approximately one-third of women were distributed across each category of education levels: no formal education (32.5 %), primary school completed (34.2 %), and secondary school completed (33.3 %). Over half of the participants (56.2 %) fell into the low category in the minimum dietary diversity score yet over half reported being food secure in the household food insecurity categories (56.1 %). The overall mean (SD) body mass index (BMI) for pregnant women at enrollment was 23.2 kg/m^2^ (4.1) and 38.4 % were nulliparous. Overall 3.1 % reported a past history of stillbirth.

A majority of the pregnant women reported iron (60.1 %) and folate (56.0 %) supplementation. The overall mean (SD) hemoglobin was 12.5 g/dl (1.9). Stratification by IRC revealed the baseline mean hemoglobin among pregnant participants in India were classified as anemic in pregnancy in both the intervention (10.3 [1.2]) and control arms (10.4 [1.3]) (WHO, 2015). In terms of household assets, 87.1 % of participant households owned a mobile phone but only 41.5 % had a bank account. While all included women were non-smokers, one of the inclusion criteria, 10.5 % reported a smoker in their household.

### 3.2. Personal exposure to PM_2.5_, BC, and CO

**Fig. 2.**
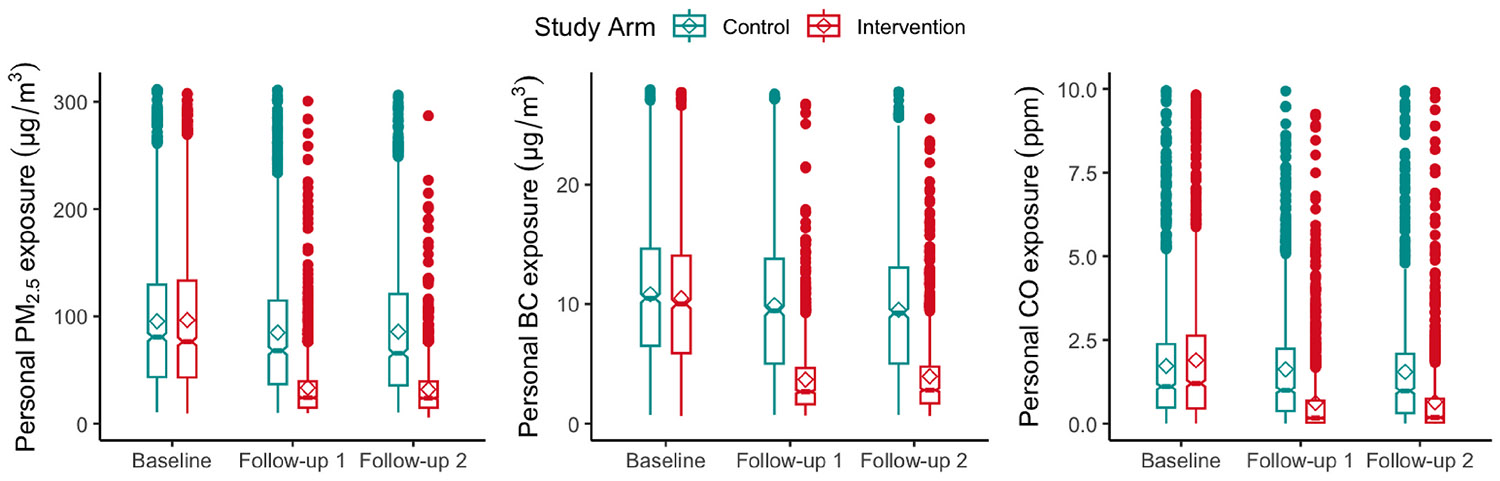
Box plots of personal PM_2.5_/BC/CO exposures over pregnancy by study arm. The diamond in each box indicates the mean value. The line is the median. The lower and upper hinges of the boxes correspond to the first and third quartiles (the 25th and 75th percentiles). The upper and lower whiskers extend 1.5 × IQR above and below the upper and lower hinges. Data points beyond the whiskers are outliers.

Among 3195 pregnant women included in the analysis, 89 % (2843), 84 % (2676), and 91 % (2904) had valid weighted exposure measurements during pregnancy for PM_2.5_, BC, and CO, respectively (Table S1). At baseline, PM_2.5_ and BC exposures were similar between the intervention and control groups, but the intervention group had statistically significantly higher CO exposures compared to the control group (Table 2 and Fig. 2), though the magnitude of this difference was relatively small (mean difference 0.42 ppm). The LPG cookstove and fuel intervention led to marked reduction in post-randomization exposures to all three pollutants in the intervention group. Notably, the median of mean personal PM_2.5_ exposure post-randomization (24.7 μg/m^3^) was below the WHO Interim Target I of 35 μg/m^3^, in the intervention group (Table 2). Detailed exposure summaries by pollutant, IRC, treatment arm and visit are presented in Supplemental Tables S2-S4.

### 3.3. Fetal and neonatal outcomes

Fetal and neonatal outcomes by study arm are presented in Table 3. Among the 3195 pregnant women in the study, due to missing outcomes, 3070 remained eligible to be included in the analysis of adverse fetal and neonatal outcomes. There were 58 stillbirths (29 intervention, 29 control), 48 congenital anomalies (23 intervention, 25 control) and 40 neonatal deaths (20 intervention, 20 control). Stillbirth, congenital anomalies, neonatal mortality and composite outcome by IRC are reported by study arm in Table 4. Across the four countries, India had the highest reported stillbirths (18), Guatemala reported the highest number of congenital anomalies (22) and Rwanda recorded the most neonatal mortalities (13).

### 3.4. ITT analysis

The results of the ITT analysis by study arm are presented in Table 3. Compared to the control arm, the relative risk among women in the intervention arm for stillbirth was 0.99 (95 % CI: 0.60, 1.66), congenital anomaly was 0.92 (95 % CI: 0.52, 1.61), and for neonatal mortality was 0.99 (95 % CI: 0.54, 1.85). The proportion of overall adverse fetal and neonatal composite outcomes (stillbirth, congenital anomaly and neonatal mortality) was 4.0 % in the intervention arm (61/1537) and 4.4 % in the control arm (67/1533), with a relative risk of 0.91 (95 % CI: 0.65, 1.28) among women randomized to the intervention for this composite outcome. Relative risks for these outcomes by IRC are reported in Table 4.

### 3.5. Exposure-Response Analysis

**Fig. 3.**
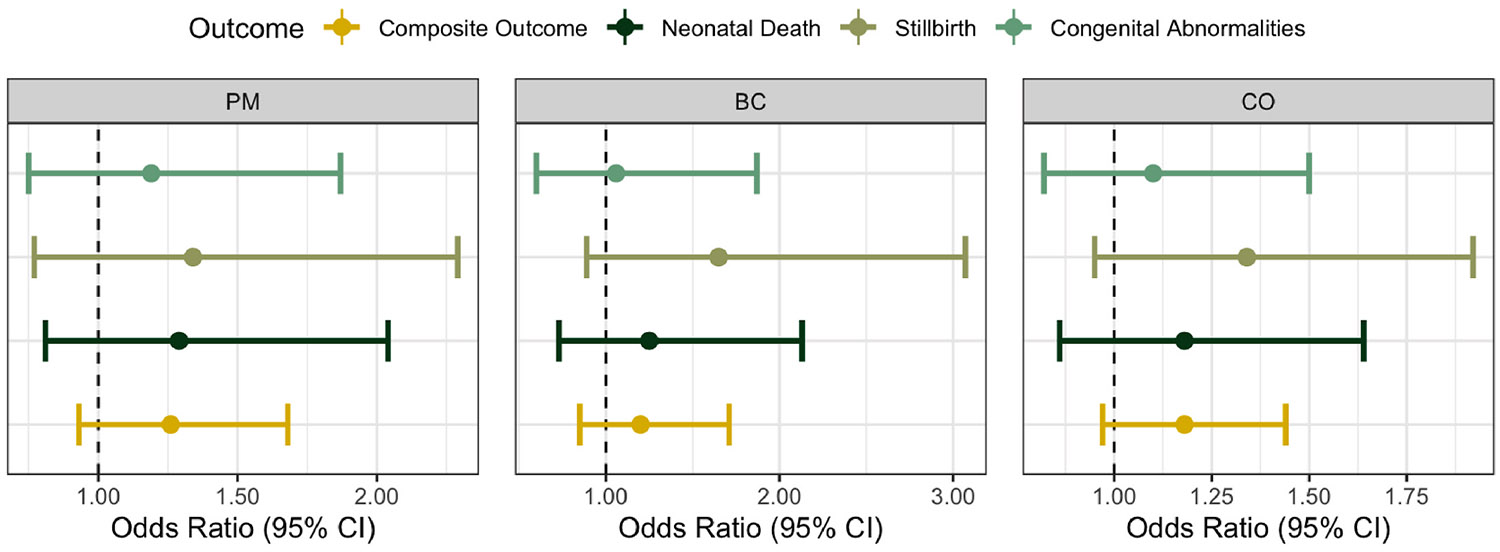
Forest plot of odds ratios and 95 % confidence intervals (CIs) based on log linear models.

Our primary exposure-response models assessed the association between stillbirths, congenital anomalies, neonatal deaths and the composite outcome and the weighted mean personal exposure to PM_2.5_, BC, and CO. We reported the adjusted odds ratios (95 % CI) of all outcomes from the log-linear exposure models in Table 5. Trial-wide crude and adjusted associations with linear and categorical exposures are shown in the Supplemental Tables S5-S6. Generally, log-linear exposure models fit better than linear and categorical exposure models trial-wide, based on the Akaike information criterion (AIC). We observed increases in odds of all outcomes of interest with 1-log-μg/m^3^ increase in PM_2.5_ and BC or with 1-log-ppm increase in CO (Fig. 3). However, none of these associations reached conventional statistical significance. The associations between the composite outcome and PM_2.5_, BC, and CO exposures resulted in narrower confidence intervals, possibly due to the larger number of cases and reduced uncertainty in the estimates. Increases in PM_2.5_ or BC exposures by 1-log-μg/m^3^ increased the odds of having any adverse fetal/neonatal outcomes by 26 % and 20 %, respectively. For a 1-log-ppm increase in CO, the odds of having any adverse fetal/neonatal (composite) outcomes in this cohort of pregnant women increased by 18 %.

For IRC-specific associations, we found a small but statistically significant association between PM_2.5_ and congenital abnormalities (adjusted odds ratio: 1.01, 95 % CI: 1.00, 1.02). In Rwanda, the odds of congenital abnormalities and stillbirth increased with higher BC exposure. The increase in personal BC exposure by every 1 μg/m^3^ increased the odds of having congenital abnormalities and stillbirth by 10 % and 6 %, respectively. Other IRC-specific adjusted associations are included in the supplemental information (Tables S7-S10). In general, they reflect the overall findings in Table 5. We did not observe any significant associations in the secondary analyses (Table S11) nor evidence of effect modification by maternal age, baseline BMI, and baseline gestational age (Table S12).

## 4. Discussion

Despite high intervention adherence and a substantial personal reductions in HAP exposure, an LPG and stove intervention did not reduce the risk of adverse fetal and neonatal outcomes (stillbirth, congenital anomaly and neonatal mortality) among pregnant women randomized to receive the intervention compared to pregnant women who continued to cook with solid fuel stoves. We observed increases in odds of all adverse fetal and neonatal outcomes of interest with higher PM_2.5_, BC, or CO exposures. Increases in PM_2.5_ or BC exposures by 1-log-μg/m^3^ increases the odds of having any adverse fetal/neonatal outcome of interest by 26 % and 20 %, respectively. For a 1-log-ppm increase in CO, the odds of having any adverse fetal/neonatal outcome of interest in this cohort of pregnant women would increase by 18 %. However, none of the associations reached conventional statistical significance (*α* = 0.05).

Previous RCTs also failed to demonstrate impacts of clean fuel interventions on adverse fetal and neonatal outcomes. Alexander et al. (2018) reported estimates of risk ratios for stillbirth (0.6, 95 % CI: 0.2, 1.9) and neonatal mortality (0.4, 95 % CI: 0.1, 1.4) among urban Nigerian women randomized to use an ethanol stove. Estimates of risk ratios for stillbirth and neonatal mortality along with several other birth outcomes comparing groups above and below the median PM_2.5_ exposures in that study also failed to reach statistical significance. A recognized limitation of the Nigerian trial was high ambient air pollution exposures that may have masked the household differences since GPS data on the pregnant women revealed that 30 % of personal exposure occurred outdoors. The Ghana Randomized Air Pollution and Health Study (GRAPHS) measured lower personal PM_2.5_ exposures among a subset of 1414 pregnant women randomized to receive an LPG stove compared to women in the control arm (continued traditional biomass use), but found no difference in the estimate of relative risk for neonatal mortality (Jack et al., 2021). While reporting on uptake of the stove intervention, the authors hypothesized that the stove intervention failed to reduce exposures enough to improve health outcomes possibly due to high housing density and/or continuing to use traditional biomass stoves alongside LPG (Jack et al., 2021). Several observational studies have reported associations between different types of household fuels and adverse fetal and neonatal outcomes. Most studies showed increased odds of stillbirth and neonatal mortality among those who used polluting fuels compared to those who used clean fuels (Younger et al., 2022). Only one study measured personal exposure to PM_2.5_ and CO (Alexander et al., 2018).

Our null findings of the effect of the HAPIN intervention on studied adverse fetal and neonatal outcomes do not seem to be attributed to inadequate exposure measurement or poor adherence to the LPG stove use. The HAPIN trial captured HAP exposure using 24-hr personal measurements of PM_2.5_, BC, and CO and the trial recruited 50–60 pregnant women a month per IRC over 12 months which would have accounted for seasonal effects (Clasen et al., 2020). The HAPIN trial exhibited high compliance of the LPG intervention using SUMS, demonstrating 96 % of participants in the intervention arm cooked exclusively with the LPG stove (Quinn et al., 2021b). The intervention group also showed a substantial, sustained reduction in exposure to PM_2.5_, BC, and CO throughout the second and third trimester of pregnancy (Johnson et al., 2022). The median post-intervention PM_2.5_ exposure was below the WHO Interim Target I for annual mean PM_2.5_ exposure of 35 μg/m^3^.

One interpretation of the findings may be that the intervention may have failed to reduce exposures sufficiently during the entire pregnancy and neonatal period to impact fetal and neonatal outcomes. Also, other compounds, such as NO_2_, that may impact adverse outcomes were not consistently measured across the four IRCs during the HAPIN trial. A recent study measured 48-hr kitchen area NO_2_ concentrations within the CHAPS trial, a RCT of an introduced LPG stove (intervention) compared to biomass stoves (controls) among 100 participants in the Peruvian Andes (Kephart et al., 2021). Results showed kitchen area NO_2_ concentration were lower within the LPG intervention arm compared to the biomass-using control arm. A review and meta-analysis by Ravindra et al. (2021) relying mainly on ambient exposure data, demonstrated prenatal exposure to PM_2.5_ and NO_2_ significantly increased the prevalence of congenital anomalies (Ravindra et al., 2021). A prospective cohort of mothers in Adama, Ethiopia found a trend towards an association between exposure to ambient NO_x_ and NO_2_ during pregnancy and increased risk of fetal death, particularly stillbirth, though the results were not statistically significant (Flanagan et al., 2022). These results suggest that there may be potential health risks associated with other fuel-related emissions, such as NO_2_.

Another potential explanation for our findings concerns the timing of the delivery of the intervention. Given that the first trimester is a crucial period for fetal development, cleaner cookstove interventions that improve household air pollution may have a larger impact if initiated early in pregnancy or even during the preconception period (Jack et al., 2021). Brain, spine, cardiac tissues begin to form in the first twelve weeks of pregnancy along with the placenta, internal organs, cartilage and limbs (ACOG, 2020). The mean gestational age for enrollment and receipt of the LPG intervention for the HAPIN trial was 15.4 weeks (SD 3.1) and the mean maternal age at baseline was 25.4 years (SD 4.5) which may explain why we did not see an intervention effect on fetal and neonatal outcomes.

Our study has several strengths. Trials are conducted in purposefully selected settings and populations; inclusion and exclusion criteria are sources of selection bias that impact external validity. We attempted to minimize these by conducting the HAPIN trial in multiple settings and introducing minimal inclusion/exclusion criteria. The chosen country sites contributed to a diverse representation of characteristics such as cooking practices, altitude, and baseline pollution exposures (Simkovich et al., 2022). The coordination by field staff, researchers and participants was executed at a high level of competency, resulting in low loss to follow-up (<5 %), high compliance with stove use and remarkable tracking of adverse outcomes even throughout the COVID-19 pandemic. Gestational age was confirmed by ultrasound and up to three 24-hr exposure assessments of three major household air pollutants: PM_2.5_, BC, and CO, per participant. To our knowledge, no study has estimated associations of BC or CO exposures with adverse fetal/neonatal outcomes nor reported exposure-response relationships with continuous exposures that allow for standard comparison across studies and generalizable risk assessment in other settings.

This study has several limitations. Since stillbirth, congenital anomalies and neonatal mortality were exploratory outcomes to the HAPIN Trial, the sample size was not calculated to detect differences in rarer fetal and neonatal outcomes presented in this study. These smaller outcome numbers restricted our analyses to exclude evaluation of subgroups. Reporting of congenital anomalies may have missed less obvious cases, such as cardiac anomalies, detected outside the study period. Pregnant women were recruited from health centers during antenatal care visits which may have biased results to better outcomes since participants were receiving antenatal care and were generally healthy non-smokers. Enrollment occurred mostly in the early second trimester, limiting the length of reduced HAP exposure and potentially missing important first trimester fetal and placental developmental windows that would have benefited from the intervention. Thus, we were not able to capture the gestational period (first trimester) when fetuses are most susceptible to formation of congenital anomalies. Additionally, since the definition of stillbirth was limited to fetal death ≥20 weeks we did not include fetal deaths occurring before 20 weeks (spontaneous abortion) in our analysis. The effect of the intervention on spontaneous abortion was reported in a separate paper (Younger et al., 2023). Field staff visited both control and intervention households and therefore were unblinded by study arm. Due to the necessity of delivering fuel to intervention households, visits from field staff may have contributed to more referrals and documenting of adverse outcomes compared to the control arm, where visits were less frequent. A number of potential covariates were missing from data collection, including the number of prenatal visits, use/compliance to new/unmonitored biomass stoves in LPG homes, or acquisition of LPG stoves in control homes. Although we have conducted more personal exposure measurements than many HAP studies (three times during pregnancy: once pre-intervention, twice post-intervention), our monitoring strategy may still have proven inadequate to fully capture exposures during the pregnancy period for a more accurate characterization of exposure-response relationship with these outcomes. For instance, some adverse events in the intervention arm were not included in the exposure-response analysis because they happened shortly after the introduction of intervention but before the first post-intervention exposure assessment. More frequent exposure measurements would have allowed us to better characterize the exposures-response analysis on each of these outcomes.

## 5. Conclusions

We did not find evidence to support a difference in stillbirth, congenital anomalies and neonatal mortality with our intervention nor did we find strong associations with HAP exposures. While the LPG intervention achieved high fidelity and adherence and demonstrated a reduction in HAP exposure in the intervention arm, our study does not provide sufficient evidence to support that these outcomes may improve with the use of an unvented LPG stove and fuel intervention. This is the first multi-country RCT using an LPG stove and fuel intervention, in which we collected detailed household air pollution exposure data and tracked adverse fetal and neonatal outcomes on pregnant women across four countries. The majority of women received the LPG intervention in the second trimester, which may be too late in fetal development to detect a protective effect. Other factors related to poverty, nutrition and access to adequate prenatal care may play a more important role in improving health outcomes. However, access to sustainable and affordable energy should remain a priority for 40 % of the global community who continue to use polluting solid fuels.

## Supplementary Material

Younger_EnvPol_2024_SI

## Figures and Tables

**Table 1:** Demographic characteristics at baseline by control and intervention arm.

	Control n = 1605	Intervention n = 1590	Overall n = 3,195^a^
**Maternal characteristics at baseline**			
Gestational week at baseline, mean (SD)	15.3 (3.2)	15.5 (3.1)	15.4 (3.1)
Maternal age, years, mean (SD)	25.4 (4.5)	25.3 (4.4)	25.4 (4.5)
<20	209 (13.0)	189 (11.9)	398 (12.5)
20–24	579 (36.1)	616 (38.7)	1195 (37.4)
25–29	517 (32.2)	500 (31.4)	1017 (31.8)
30–35	300 (18.7)	285 (17.9)	585 (18.3)
Highest level of education achieved, n (%)			
No formal education	558 (34.8)	481 (30.3)	1039 (32.5)
Primary completed	533 (33.2)	558 (35.1)	1091 (34.1)
Secondary completed	514 (32.0)	550 (34.6)	1064 (33.3)
Missing	0 (0.0)	1 (0.1)	1 (0.0)
Minimum dietary diversity^b^, category (score), n (%)			
Low (<4)	906 (56.4)	890 (56.0)	1796 (56.2)
Medium (4–5)	533 (33.2)	496 (31.2)	1029 (32.2)
High (>5)	165 (10.3)	203 (12.8)	368 (11.5)
Missing	1 (0.1)	1 (0.1)	2 (0.1)
Household food insecurity^c^, category (score), n (%)			
Food secure (0)	863 (53.8)	930 (58.5)	1793 (56.1)
Mild (1, 2, 3)	448 (27.9)	416 (26.2)	864 (27.0)
Moderate (4, 5, 6)/Severe (7, 8)	272 (16.9)	220 (13.8)	492 (15.4)
Missing	22 (1.4)	24 (1.5)	46 (1.4)
Body mass index (BMI), kg/m^2^, mean (SD); n missing	23.1 (4.0); 7	23.3 (4.1); 12	23.2 (4.1); 19
Maternal hemoglobin level, grams per Liter (g/dL); mean (SD); n missing	12.5 (1.9); 13	12.4 (1.9); 17	12.5 (1.9); 30
Vitamin Intake^d^, n (%)			
Multiple micronutrient tablets	198 (12.3)	181 (11.4)	379 (11.9)
Iron	974 (60.7)	947 (59.6)	1921 (60.1)
Vitamin A	15 (0.9)	10 (0.6)	25 (0.8)
Folate	911 (56.8)	877 (55.2)	1788 (56.0)
Other	46 (2.9)	44 (2.8)	90 (2.8)
None	314 (19.6)	342 (21.5)	656 (20.5)
Nulliparous^e^, mean (SD)			
Yes	589 (36.7)	639 (40.2)	1228 (38.4)
No	1014 (63.2)	947 (59.6)	1961 (61.4)
Missing	2 (0.1)	4 (0.3)	6 (0.2)
Reported history of stillbirth, n (%)			
Yes	44 (2.7)	56 (3.5)	100 (3.1)
No	1561 (97.3)	1534 (96.5)	3095 (96.9)
**Exposure characteristics**			
Someone in household smokes^f^, n (%)			
Yes	181 (11.3)	153 (9.6)	334 (10.5)
No	1421 (88.5)	1436 (90.3)	2857 (89.4)
Missing	3 (0.2)	1 (0.1)	4 (0.1)
**Household characteristics**			
Number of people sleeping in house, mean (SD);	4.3 (2.0)	4.3 (2.0)	4.3 (2.0)
Owns household assets, n (%)			
Color Television	783 (48.8)	774 (48.7)	1557 (48.7)
Radio	721 (44.9)	734 (46.2)	1455 (45.5)
Mobile phone	1,395 (86.9)	1388 (87.3)	2783 (87.1)
Bicycle	409 (25.5)	365 (23.0)	774 (24.2)
Bank account	628 (39.1)	697 (43.8)	1325 (41.5)

a N = 3200 women were randomized; 5 women were deemed ineligible after randomization.

b Adapted from Food and Agriculture Organization of the United Nations Minimum Diet Diversity for Women (FAO 2016b).

c The Food Insecurity Experience Scale, developed by the Food and Agriculture Organization of the United Nations, http://www.fao.org/3/as583e/as583e.pdf.

d Vitamins taken in the past 12 months.

e Nulliparous defined as zero pregnancies reaching 20 weeks and 0 days of gestation or beyond; miscarriages can have occurred in a woman who is nulliparous.

f Someone in the household other than the pregnant woman smokes; pregnant women were all non-smokers based on eligibility criteria.

**Table 2:** Summary of personal exposure to PM_2.5_, BC, and CO by study arm and visit.

IRC	Arm	N^a^	Mean (SD)	Median (IQR)	Range
***Baseline***					
PM_2.5_	Control	1422	111 (110)	83.1 (45.9–141)	10.5–1799
	Intervention	1401	120 (135)	81.7 (45.9–151)	9.36–2100
BC	Control	1272	12.4 (9.43)	10.8 (6.81–15.5)	0.72–95.6
	Intervention	1267	12.6 (11)	10.5 (6.2–15.3)	0.636–133
CO	Control	1447	2.3 (3.97)	1.18 (0.502–2.53)	0–60.2
	Intervention	1430	2.72 (4.75)	1.32 (0.482–2.99)	0–69.5
***Follow-up 1***					
PM_2.5_	Control	1251	104 (114)	71.5 (38.5–126)	9.89–1117
	Intervention	1285	33.8 (33.1)	24.1 (15–39.5)	9.59–459
BC	Control	1187	11.1 (9.56)	9.73 (5.28–14.4)	0.722–122
	Intervention	1226	3.97 (5.47)	2.68 (1.62–4.71)	0.666–131
CO	Control	1311	2.25 (4.06)	1.06 (0.396–2.5)	0–64.2
	Intervention	1315	0.687 (1.53)	0.172 (0.0315–0.699)	0–23.9
***Follow-up 2***					
PM_2.5_	Control	1138	102 (108)	69.5 (36.5–131)	10.2–1208
	Intervention	1176	35.8 (54.6)	23.7 (14.9–39.7)	5.7–851
BC	Control	1079	11.1 (10.2)	9.57 (5.21–13.7)	0.72–124
	Intervention	1134	4.28 (5.44)	2.82 (1.69–4.83)	0.635–105
CO	Control	1213	2.21 (3.98)	1.06 (0.333–2.29)	0–43.7
	Intervention	1227	0.668 (1.34)	0.184 (0.0329–0.749)	0–21.2

a Summary based on valid exposure measurements.

**Table 3:** Effect of the intervention on stillbirth, congenital anomalies, neonatal mortality and composite outcome.^a^.

Outcome	Intervention n (%)	Control n (%)	Relative Risk^b^ (95% CI)
Stillbirth			
Yes, n (%)	29 (1.9)	29 (1.9)	0.99 (0.60, 1.66)
No, n (%)	1508 (98.1)	1504 (98.1)	
Congenital Anomaly			
Yes, n (%)	23 (1.5)	25 (1.6)	0.92 (0.52, 1.61)
No, n (%)	1514 (98.5)	1508 (98.4)	
Neonatal Mortality			
Yes, n (%)	20 (1.3)	20 (1.3)	0.99 (0.54, 1.85)
No, n (%)	1488 (98.7)	1484 (98.7)	
Composite Outcome			
Yes, n (%)	61 (4.0)	67 (4.4)	0.91 (0.65, 1.28)
No, n (%)	1476 (96.0)	1466 (95.6)	

a Denominator for congenital anomaly, stillbirth and composite was 3080. Denominator for neonatal mortality was 3012.

b Relative risk reported as intervention compared to control.

**Table 4:** Effects of the intervention on stillbirth, congenital anomalies, neonatal mortality and composite outcome by country site.

Outcome	Guatemala		India		Peru		Rwanda	
	I (n = 390)	C (n = 389)	I (n = 395)	C (n = 393)	I (n = 378)	C (n = 358)	I (n = 374)	C (n = 393)
*Stillbirth*								
Yes, n (%)	10 (2.6)	5 (1.3)	10 (2.5)	8 (2.0)	2 (0.5)	7 (2.0)	7 (1.9)	9 (2.3)
No, n (%)	380 (97.4)	385 (98.7)	385 (97.5)	385 (98.0)	376 (99.5)	351 (98.0)	387 (98.1)	384 (97.7)
Relative Risk (95 % CI) *Congenital Anomaly*	1.99 (0.69, 5.78)		1.24 (0.50, 3.12)		0.27 (0.06, 1.29)		0.82 (0.31, 2.17)	
Yes, n (%)	9 (2.3)	13 (3.3)	7 (1.8)	5 (1.3)	5 (1.3)	3 (0.7)	2 (0.5)	4 (1.0)
No, n (%)	381 (97.7)	376 (96.7)	388 (98.2)	388 (98.7)	373 (98.7)	355 (99.2)	372 (99.5)	389 (99.0)
Relative Risk (95 % CI)	0.69 (0.30, 1.60)		1.39 (0.45, 4.35)		1.57 (0.38, 6.56)		0.55 (0.10, 2.85)	
*Neonatal Mortality*								
Yes, n (%)	5 (1.3)	6 (1.6)	4 (1.0)	3 (0.8)	4 (1.1)	5 (1.4)	7 (1.9)	6 (1.6)
No, n (%)	375 (98.7)	378 (98.4)	381 (99.0)	382 (99.2)	372 (98.9)	346 (98.6)	360 (98.1)	378 (98.4)
Relative Risk (95 % CI)	0.84 (0.26, 2.74)		1.33 (0.30, 5.92)		0.75 (0.20, 2.76)		1.22 (0.41, 3.60)	
*Composite Outcome*								
Yes, n (%)	20 (5.1)	21 (5.4)	17 (4.3)	14 (3.6)	9 (2.4)	14 (3.9)	15 (4.0)	18 (4.6)
No, n (%)	370 (94.9)	368 (94.6)	378 (95.7)	379 (96.4)	369 (97.6)	344 (96.1)	359 (96.0)	375 (95.4)
Relative Risk (95 % CI)	0.95 (0.52, 1.72)		1.21 (0.60, 2.42)		0.61 (0.27, 1.39)		0.87 (0.45, 1.71)	

**Table 5:** Exposure-response results between weighted mean PM_2.5_, BC and CO exposures and on congenital anomalies, stillbirth, neonatal mortality and composite outcomes.^a^.

Exposures	Odds Ratio	95 % CI	p-value	AIC
*Stillbirth*				
PM_2.5_	1.34	(0.77, 2.29)	0.29	308
BC	1.65	(0.89, 3.07)	0.11	295
CO	1.34	(0.95, 1.92)	0.10	326
*Congenital Anomaly*				
PM_2.5_	1.19	(0.75, 1.87)	0.44	407
BC	1.06	(0.60, 1.87)	0.83	387
CO	1.10	(0.82, 1.50)	0.52	429
*Neonatal Death*				
PM_2.5_	1.29	(0.81, 2.04)	0.28	403
BC	1.25	(0.73, 2.13)	0.42	392
CO	1.18	(0.86, 1.64)	0.31	388
*Composite Outcomes*				
PM_2.5_	1.26	(0.93, 1.68)	0.13	811
BC	1.20	(0.85, 1.71)	0.30	773
CO	1.18	(0.97, 1.44)	0.10	822

a All models adjusted for IRC (country), maternal age at baseline, nulliparity, mother’s education, BMI at baseline, hemoglobin level at baseline, household food insecurity, mother’s diet diversity and whether there was a smoker presented at home.

## Data Availability

Data will be made available on request.

## Appendix A. Supplementary data

Supplementary data to this article can be found online at https://doi.org/10.1016/j.envpol.2024.123414.
