# Supplementary material for: Effects of a liquefied petroleum gas stove intervention on stillbirth, congenital anomalies and neonatal mortality: A multi-country household air pollution intervention network trial
Source: Environ Pollut. Author manuscript; Available in PMC 2026 Jun 25. (PMC13296888; doi:10.1016/j.envpol.2024.123414)
Supplement: Younger_EnvPol_2024_SI [file NIHMS2165622-supplement-Younger_EnvPol_2024_SI.docx]

**Supplemental Information**

| **Table S1.** Weighted mean exposure of PM_2.5_, BC, and CO by treatment arm and IRC |
| --- |
| **Table S2.** Summary of personal exposure to PM_2.5_ by treatment arm, IRC, and visit |
| **Table S3.** Summary of personal exposure to BC by treatment arm, IRC, and visit |
| **Table S4**. Summary of personal exposure to CO by treatment arm, IRC, and visit |
| **Table S5.** Exposure-response results between weighted mean PM_2.5_/BC/CO exposures and fetal/neonatal outcomes (linear and categorical exposure models). |
| **Table S6**. Crude associations between weighted mean PM_2.5_/BC/CO exposures and fetal/neonatal outcomes. |
| **Table S7**. Adjusted associations between weighted mean PM_2.5_/BC/CO exposures and fetal/neonatal outcomes in Guatemala |
| **Table S8**. Adjusted associations between weighted mean PM_2.5_/BC/CO exposures and fetal/neonatal outcomes in India |
| **Table S9**. Adjusted associations between weighted mean PM_2.5_/BC/CO exposures and fetal/neonatal outcomes in Peru |
| **Table S10**. Adjusted associations between weighted mean PM_2.5_/BC/CO exposures and fetal/neonatal outcomes in Rwanda |
| **Table S11**. Adjusted associations between mean post-intervention PM_2.5_/BC/CO exposures and fetal/neonatal outcomes. |
| **Table S12**. Assessment of effect modification by maternal age, baseline BMI and baseline gestational age |
| **Figure S1.** Box plots of weighted mean PM_2.5_/BC/CO exposures over pregnancy by arm. The square in each box indicates the mean value. |

**Table S1**. Weighted mean exposure of PM_2.5_, BC, and CO by treatment arm and IRC

| **IRC** | **Arm** | **N** | **Mean (SD)** | **Median (IQR)** | **Range** |
| --- | --- | --- | --- | --- | --- |
| ***PM2.5*** | | | | | |
| Guatemala | Control | 400 | 135 (93.7) | 112 (70.1 - 180) | 15.0 - 792 |
|  | Intervention | 400 | 87.2 (61.7) | 69.3 (44.8 - 112) | 15.0 - 476 |
| India | Control | 399 | 106 (87.3) | 79.7 (50.1 - 131) | 15.8 - 593 |
|  | Intervention | 400 | 81.7 (94.9) | 55.9 (38.7 - 92.2) | 10.7 - 872 |
| Peru | Control | 402 | 75.1 (94.0) | 44.0 (22.8 - 91.3) | 10.7 - 723 |
|  | Intervention | 396 | 51.5 (63.1) | 34.1 (20.7 - 58.9) | 11.8 - 782 |
| Rwanda | Control | 404 | 111 (83.2) | 92.1 (63.9 - 133) | 14.2 - 1090 |
|  | Intervention | 394 | 72.9 (48.3) | 61.0 (39.8 - 89.8) | 18.4 - 364 |
| All | Control | 1605 | 107 (92.0) | 83.1 (47.4 - 135) | 10.7 - 1090 |
|  | Intervention | 1590 | 74.4 (70.5) | 55.3 (35.0 - 91.9) | 10.7 - 872 |
| ***BC*** | | | | | |
| Guatemala | Control | 400 | 12.5 (5.3) | 11.8 (9.78 - 14.5) | 2.6 - 59.3 |
|  | Intervention | 400 | 8.9 (6.9) | 7.9 (6.4 - 9.7) | 2.6 - 82.9 |
| India | Control | 399 | 11.9 (8.8) | 9.6 (6.0 - 14.9) | 0.8 - 78.5 |
|  | Intervention | 400 | 8.7 (7.71) | 6.7 (4.1 - 11.2) | 1.2 - 66.1 |
| Peru | Control | 402 | 9.5 (9.8) | 6.9 (2.71 - 12.5) | 1.41 - 69.7 |
|  | Intervention | 396 | 6.0 (4.5) | 4.7 (2.8 - 7.8) | 1.5 - 26.1 |
| Rwanda | Control | 404 | 12.1 (7.1) | 11.1 (8.1 - 14.1) | 2.8 - 65.7 |
|  | Intervention | 394 | 8.1 (4.6) | 7.0 (5.1 - 9.3) | 2.8 - 43.5 |
| All | Control | 1605 | 11.5 (8.0) | 10.5 (6.6 - 14.2) | 0.8 - 78.5 |
|  | Intervention | 1590 | 8.0 (6.3) | 6.9 (4.5 - 9.5) | 1.2 - 82.9 |
| ***CO*** | | | | | |
| Guatemala | Control | 400 | 1.94 (2.14) | 1.39 (0.74 - 2.41) | 0.04 - 22.3 |
|  | Intervention | 400 | 1.20 (1.20) | 0.86 (0.49 - 1.51) | 0 - 11.8 |
| India | Control | 399 | 1.90 (2.66) | 1.07 (0.51 - 2.14) | 0 - 26.2 |
|  | Intervention | 400 | 1.05 (1.42) | 0.62 (0.25 - 1.34) | 0 - 18.0 |
| Peru | Control | 402 | 3.45 (5.06) | 1.80 (0.84 - 3.85) | 0 - 46.4 |
|  | Intervention | 396 | 2.66 (3.43) | 1.66 (0.83 - 2.97) | 0.01 - 31.4 |
| Rwanda | Control | 404 | 2.15 (2.87) | 1.25 (0.70 - 2.26) | 0.02 - 23.9 |
|  | Intervention | 394 | 1.65 (2.24) | 0.92 (0.45 - 1.85) | 0.01 - 22.6 |
| All | Control | 1605 | 2.35 (3.42) | 1.34 (0.67 - 2.63) | 0 - 46.4 |
|  | Intervention | 1590 | 1.57 (2.22) | 0.94 (0.45 - 1.82) | 0 - 31.4 |

*Note: Summary based on valid exposure measurements.*

**Table S2**. Summary of personal exposure to PM_2.5_ by treatment arm, IRC, and visit

| **IRC** | **Arm** | **N** | **Mean (SD)** | **Median (IQR)** | **Range** |
| --- | --- | --- | --- | --- | --- |
| ***Baseline*** | | | | | |
| Guatemala | Control | 373 | 140 (133) | 110 (63.3 - 176) | 10.5 - 1799 |
|  | Intervention | 360 | 153 (119) | 122 (65.8 - 200) | 9.94 - 780 |
| India | Control | 358 | 104 (100) | 73.8 (46.7 - 123) | 10.8 - 1034 |
|  | Intervention | 357 | 127 (181) | 78.4 (47.9 - 143) | 9.36 - 2100 |
| Peru | Control | 327 | 80.2 (94.8) | 46.1 (15.2 - 108) | 10.7 - 697 |
|  | Intervention | 331 | 89.5 (117) | 53.3 (22.8 - 111) | 11 - 1400 |
| Rwanda | Control | 364 | 116 (97.2) | 94 (58.6 - 145) | 14.3 - 1090 |
|  | Intervention | 353 | 108 (98.4) | 82.7 (47.8 - 134) | 14.2 - 866 |
| All | Control | 1422 | 111 (110) | 83.1 (45.9 - 141) | 10.5 - 1799 |
|  | Intervention | 1401 | 120 (135) | 81.7 (45.9 - 151) | 9.36 - 2100 |
| ***Follow-up 1*** | | | | | |
| Guatemala | Control | 339 | 133 (116) | 98.4 (58.7 - 165) | 9.89 - 682 |
|  | Intervention | 361 | 31.3 (33.4) | 23.3 (14.9 - 36.2) | 9.59 - 459 |
| India | Control | 311 | 103 (115) | 67.3 (38.9 - 118) | 10.5 - 890 |
|  | Intervention | 314 | 39.2 (39.1) | 28.7 (16.9 - 45.5) | 10.4 - 301 |
| Peru | Control | 269 | 64.5 (104) | 31.4 (14.5 - 74.4) | 9.9 - 1117 |
|  | Intervention | 289 | 20.8 (19.3) | 14.6 (14.1 - 23.1) | 9.65 - 259 |
| Rwanda | Control | 332 | 109 (110) | 79.6 (48.5 - 130) | 14 - 1093 |
|  | Intervention | 321 | 43.1 (32.3) | 33.6 (23.8 - 50) | 14.2 - 284 |
| All | Control | 1251 | 104 (114) | 71.5 (38.5 - 126) | 9.89 - 1117 |
|  | Intervention | 1285 | 33.8 (33.1) | 24.1 (15 - 39.5) | 9.59 - 459 |
| ***Follow-up 2*** | | | | | |
| Guatemala | Control | 317 | 124 (99.5) | 93.6 (53.6 - 168) | 10.3 - 689 |
|  | Intervention | 330 | 33.4 (37.9) | 23.8 (16.3 - 38.5) | 9.74 - 442 |
| India | Control | 284 | 109 (123) | 68.2 (36.3 - 129) | 10.4 - 794 |
|  | Intervention | 293 | 36.5 (39.6) | 25.3 (16.9 - 41.8) | 5.7 - 463 |
| Peru | Control | 219 | 67 (125) | 24.7 (14.5 - 57.9) | 10.2 - 1208 |
|  | Intervention | 264 | 28.1 (77.1) | 14.6 (13.9 - 18.6) | 10.5 - 851 |
| Rwanda | Control | 318 | 99.4 (77.9) | 79.9 (45.9 - 128) | 14.6 - 665 |
|  | Intervention | 289 | 45 (58.1) | 28.2 (23.6 - 49.1) | 12.5 - 752 |
| All | Control | 1138 | 102 (108) | 69.5 (36.5 - 131) | 10.2 - 1208 |
|  | Intervention | 1176 | 35.8 (54.6) | 23.7 (14.9 - 39.7) | 5.7 - 851 |

*Note: Summary based on valid exposure measurements.*

**Table S3**. Summary of personal exposure to BC by treatment arm, IRC, and visit

| **IRC** | **Arm** | **N** | **Mean (SD)** | **Median (IQR)** | **Range** |
| --- | --- | --- | --- | --- | --- |
| ***Baseline*** | | | | | |
| Guatemala | Control | 343 | 13 (7.49) | 12.1 (9.12 - 15.3) | 2.55 -95.6 |
|  | Intervention | 332 | 13.4 (10.7) | 11.7 (9.36 - 14.8) | 2.55 -133 |
| India | Control | 351 | 12.3 (9.9) | 9.39 (5.55 - 15.7) | 0.72 -73.2 |
|  | Intervention | 348 | 13.5 (12.6) | 9.77 (5.49 - 16.4) | 0.636 -103 |
| Peru | Control | 289 | 11 (11.6) | 7.89 (2.31 - 15.7) | 1.46 -74.9 |
|  | Intervention | 307 | 11.6 (11.2) | 8.74 (3.6 - 15.5) | 1.48 -75.3 |
| Rwanda | Control | 289 | 13 (8.37) | 11.8 (8.27 - 15.6) | 2.66 -70.4 |
|  | Intervention | 280 | 11.5 (8.75) | 9.67 (6.57 - 14.2) | 2.71 -76.9 |
| All | Control | 1272 | 12.4 (9.43) | 10.8 (6.81 - 15.5) | 0.72 -95.6 |
|  | Intervention | 1267 | 12.6 (11) | 10.5 (6.2 - 15.3) | 0.636 -133 |
| ***Follow-up 1*** | | | | | |
| Guatemala | Control | 330 | 12.3 (6.63) | 11.5 (8.1 - 15.1) | 2.57 -52.9 |
|  | Intervention | 359 | 4.89 (7.51) | 2.7 (2.61 - 5.34) | 2.23 -131 |
| India | Control | 304 | 11.1 (10.3) | 8.85 (4.55 - 14.5) | 0.722 -98.5 |
|  | Intervention | 305 | 3.48 (4.67) | 2.11 (1.13 - 3.6) | 0.666 -44.6 |
| Peru | Control | 246 | 8.5 (10.1) | 4.37 (1.59 - 12.1) | 1.4 -73.6 |
|  | Intervention | 268 | 1.89 (1.15) | 1.58 (1.54 - 1.63) | 1.37 -13.8 |
| Rwanda | Control | 307 | 11.8 (10.6) | 10 (7 - 13.9) | 2.81 -122 |
|  | Intervention | 294 | 5.27 (5.04) | 4.21 (2.92 - 5.87) | 2.63 -54.7 |
| All | Control | 1187 | 11.1 (9.56) | 9.73 (5.28 - 14.4) | 0.722 -122 |
|  | Intervention | 1226 | 3.97 (5.47) | 2.68 (1.62 - 4.71) | 0.666 -131 |
| ***Follow-up 2*** | | | | | |
| Guatemala | Control | 310 | 11.9 (7.14) | 11.1 (8.37 - 14.5) | 2.52 -88.1 |
|  | Intervention | 326 | 4.99 (4.9) | 2.88 (2.61 - 5.66) | 2.52 -64.8 |
| India | Control | 277 | 11.8 (12.3) | 8.24 (4.38 - 14) | 0.72 -97.8 |
|  | Intervention | 289 | 4.24 (7.57) | 2.48 (1.52 - 4.06) | 0.635 -105 |
| Peru | Control | 201 | 8.65 (13.8) | 3.65 (1.59 - 10.8) | 1.33 -124 |
|  | Intervention | 242 | 2.02 (1.77) | 1.58 (1.55 - 1.63) | 1.35 -14.5 |
| Rwanda | Control | 291 | 11.2 (7.15) | 10.3 (6.41 - 13.7) | 2.82 -61.5 |
|  | Intervention | 277 | 5.47 (4.9) | 3.81 (2.91 - 6.18) | 2.5 -44 |
| All | Control | 1079 | 11.1 (10.2) | 9.57 (5.21 - 13.7) | 0.72 -124 |
|  | Intervention | 1134 | 4.28 (5.44) | 2.82 (1.69 - 4.83) | 0.635 -105 |

*Note: Summary based on valid exposure measurements.*

**Table S4**. Summary of personal exposure to CO by treatment arm, IRC, and visit

| **IRC** | **Arm** | **N** | **Mean (SD)** | **Median (IQR)** | **Range** |
| --- | --- | --- | --- | --- | --- |
| ***Baseline*** | | | | | |
| Guatemala | Control | 382 | 2.01 (3.68) | 1.29 (0.55 - 2.48) | 0 - 60.2 |
|  | Intervention | 375 | 2.06 (2.28) | 1.37 (0.61 - 2.76) | 0 - 21.7 |
| India | Control | 372 | 1.8 (3.64) | 0.80 (0.33 - 1.91) | 0 - 46.9 |
|  | Intervention | 373 | 1.71 (2.6) | 0.89 (0.32 - 2.15) | 0 - 31.6 |
| Peru | Control | 333 | 3.47 (4.95) | 1.87 (0.74 - 4.30) | 0 - 54.3 |
|  | Intervention | 326 | 4.38 (7.49) | 1.93 (0.76 - 4.80) | 0 - 69.5 |
| Rwanda | Control | 360 | 2.03 (3.34) | 1.05 (0.54 - 1.99) | 0 - 29.6 |
|  | Intervention | 356 | 2.95 (4.79) | 1.18 (0.48 - 3.04) | 0 - 44.4 |
| All | Control | 1447 | 2.3 (3.97) | 1.18 (0.50 - 2.53) | 0 - 60.2 |
|  | Intervention | 1430 | 2.72 (4.75) | 1.32 (0.48 - 2.99) | 0 - 69.5 |
| ***Follow-up 1*** | | | | | |
| Guatemala | Control | 355 | 1.91 (2.21) | 1.22 (0.52 - 2.52) | 0 - 21.7 |
|  | Intervention | 363 | 0.46 (0.98) | 0.15 (0.04 - 0.47) | 0 - 11.9 |
| India | Control | 352 | 1.9 (3.22) | 0.78 (0.23 - 2.11) | 0 - 24.7 |
|  | Intervention | 347 | 0.44 (1.36) | 0.03 (0.00 - 0.26) | 0 - 17.3 |
| Peru | Control | 269 | 3.34 (6.74) | 1.23 (0.33 - 3.37) | 0 - 64.2 |
|  | Intervention | 264 | 1.41 (2.36) | 0.67 (0.20 - 1.75) | 0 - 23.9 |
| Rwanda | Control | 335 | 2.08 (3.29) | 1.00 (0.42 - 2.14) | 0 - 25.9 |
|  | Intervention | 341 | 0.63 (1.15) | 0.20 (0.07 - 0.70) | 0 - 9.25 |
| All | Control | 1311 | 2.25 (4.06) | 1.06 (0.40 - 2.50) | 0 - 64.2 |
|  | Intervention | 1315 | 0.67 (1.53) | 0.17 (0.03 - 0.70) | 0 - 23.9 |
| ***Follow-up 2*** | | | | | |
| Guatemala | Control | 336 | 1.70 (2.08) | 1.16 (0.40 - 2.16) | 0 - 14.4 |
|  | Intervention | 344 | 0.57 (1.07) | 0.17 (0.04 - 0.64) | 0 - 8.90 |
| India | Control | 320 | 1.96 (3.75) | 0.74 (0.15 - 2.23) | 0 - 35.9 |
|  | Intervention | 313 | 0.38 (0.87) | 0.04 (0.00 - 0.25) | 0 - 6.85 |
| Peru | Control | 212 | 3.46 (6.28) | 1.46 (0.48 - 3.31) | 0 - 43.7 |
|  | Intervention | 250 | 1.21 (2.15) | 0.56 (0.15 - 1.30) | 0 - 21.2 |
| Rwanda | Control | 345 | 2.15 (3.63) | 1.06 (0.45 - 2.16) | 0 - 26.4 |
|  | Intervention | 320 | 0.63 (0.99) | 0.22 (0.07 - 0.74) | 0 - 6.74 |
| All | Control | 1213 | 2.21 (3.98) | 1.06 (0.33 - 2.29) | 0 - 43.7 |
|  | Intervention | 1227 | 0.67 (1.34) | 0.18 (0.03 - 0.75) | 0 - 21.2 |

*Note: Summary based on valid exposure measurements.*

**Table S5**. Exposure-response results between weighted mean PM_2.5_/BC/CO exposures and fetal/neonatal outcomes (linear and categorical exposure models).

| **Exposures** | **Model Type** | **Odds Ratio** | **95% CI** | **p-value** | **AIC** |
| --- | --- | --- | --- | --- | --- |
| *Congenital Abnormalities* | |  |  |  |  |
| PM_2.5_ | Linear | 1.00 | (1.00, 1.00) | 0.20 | 406 |
|  | Categorical [Ref. Quartile 1] (n = 10) |  |  |  |  |
|  | Quartile 2 (n = 6) | 0.54 | (0.18, 1.48) | 0.24 | 409 |
|  | Quartile 3 (n = 10) | 0.96 | (0.38, 2.41) | 0.92 | 409 |
|  | Quartile 4 (n = 12) | 1.12 | (0.46, 2.74) | 0.81 | 409 |
| BC | Linear | 1.01 | (0.97, 1.05) | 0.48 | 387 |
|  | Categorical [Ref. Quartile 1] (n = 9) |  |  |  |  |
|  | Quartile 2 (n = 11) | 1.13 | (0.46, 2.86) | 0.79 | 390 |
|  | Quartile 3 (n = 7) | 0.70 | (0.24, 1.93) | 0.49 | 390 |
|  | Quartile 4 (n = 9) | 0.91 | (0.34, 2.42) | 0.85 | 390 |
| CO | Linear | 0.98 | (0.83, 1.09) | 0.82 | 429 |
|  | Categorical [Ref. Quartile 1] (n = 8) |  |  |  |  |
|  | Quartile 2 (n = 16) | 1.92 | (0.83, 4.78) | 0.14 | 429 |
|  | Quartile 3 (n = 6) | 0.74 | (0.24, 2.15) | 0.58 | 429 |
|  | Quartile 4 (n = 10) | 1.18 | (0.46, 3.15) | 0.73 | 429 |
| *Stillbirth* |  |  |  |  |  |
| PM_2.5_ | Linear | 1.00 | (1.00, 1.00) | 0.27 | 308 |
|  | Categorical [Ref. Quartile 1] (n = 3) |  |  |  |  |
|  | Quartile 2 (n = 10) | 3.77 | (1.13, 17.0) | 0.05 | 307 |
|  | Quartile 3 (n = 5) | 1.41 | (0.31, 7.28) | 0.66 | 307 |
|  | Quartile 4 (n = 8) | 3.01 | (0.84, 14.1) | 0.11 | 307 |
| BC | Linear | 1.03 | (0.99, 1.07) | 0.07 | 295 |
|  | Log linear | 1.65 | (0.89, 3.07) | 0.11 | 295 |
|  | Categorical [Ref. Quartile 1] (n = 2) |  |  |  |  |
|  | Quartile 2 (n = 10) | 5.46 | (1.41, 35.9) | 0.03 | 295 |
|  | Quartile 3 (n = 7) | 3.31 | (0.75, 23.0) | 0.15 | 295 |
|  | Quartile 4 (n = 6) | 3.31 | (0.74, 23.1) | 0.15 | 295 |
| CO | Linear | 1.06 | (0.96, 1.13) | 0.13 | 327 |
|  | Categorical [Ref. Quartile 1] (n = 7) |  |  |  |  |
|  | Quartile 2 (n = 3) | 0.27 | (0.04, 1.13) | 0.11 | 325 |
|  | Quartile 3 (n = 6) | 0.84 | (0.27, 2.55) | 0.75 | 325 |
|  | Quartile 4 (n = 12) | 1.63 | (0.65, 4.43) | 0.31 | 325 |
| *Neonatal Death* | |  |  |  |  |
| PM_2.5_ | Linear | 1.00 | (1.00, 1.00) | 0.72 | 404 |
|  | Categorical [Ref. Quartile 1] (n = 7) |  |  |  |  |
|  | Quartile 2 (n = 7) | 0.96 | (0.32, 2.86) | 0.94 | 406 |
|  | Quartile 3 (n = 11) | 1.59 | (0.61, 4.43) | 0.36 | 406 |
|  | Quartile 4 (n = 13) | 1.68 | (0.65, 4.68) | 0.29 | 406 |
| BC | Linear | 1.01 | (0.96, 1.05) | 0.60 | 393 |
|  | Categorical [Ref. Quartile 1] (n = 7) |  |  |  |  |
|  | Quartile 2 (n = 9) | 1.25 | (0.46, 3.57) | 0.66 | 394 |
|  | Quartile 3 (n = 8) | 0.98 | (0.33, 2.93) | 0.97 | 394 |
|  | Quartile 4 (n = 13) | 1.86 | (0.73, 5.09) | 0.20 | 394 |
| CO | Linear | 1.00 | (0.84, 1.10) | 0.95 | 389 |
|  | Categorical [Ref. Quartile 1] (n = 5) |  |  |  |  |
|  | Quartile 2 (n = 12) | 2.22 | (0.81, 7.07) | 0.14 | 389 |
|  | Quartile 3 (n = 6) | 1.21 | (0.36, 4.23) | 0.76 | 389 |
|  | Quartile 4 (n = 13) | 2.30 | (0.84, 7.33) | 0.12 | 389 |
| *Composite Outcomes* | |  |  |  |  |
| PM_2.5_ | Linear | 1.00 | (1.00, 1.00) | 0.18 | 812 |
|  | Categorical [Ref. Quartile 1] (n = 18) |  |  |  |  |
|  | Quartile 2 (n = 21) | 1.16 | (0.61, 2.23) | 0.66 | 815 |
|  | Quartile 3 (n = 24) | 1.28 | (0.68, 2.46) | 0.44 | 815 |
|  | Quartile 4 (n = 29) | 1.53 | (0.83, 2.88) | 0.18 | 815 |
| BC | Linear | 1.01 | (0.98, 1.04) | 0.36 | 773 |
|  | Categorical [Ref. Quartile 1] (n = 17) |  |  |  |  |
|  | Quartile 2 (n = 27) | 1.58 | (0.86, 3.01) | 0.15 | 774 |
|  | Quartile 3 (n = 19) | 0.97 | (0.48, 1.95) | 0.93 | 774 |
|  | Quartile 4 (n = 25) | 1.43 | (0.76, 2.77) | 0.27 | 774 |
| CO | Linear | 1.02 | (0.93, 1.08) | 0.67 | 824 |
|  | Categorical [Ref. Quartile 1] (n = 17) |  |  |  |  |
|  | Quartile 2 (n = 29) | 1.63 | (0.89, 3.07) | 0.12 | 822 |
|  | Quartile 3 (n = 16) | 0.95 | (0.47, 1.91) | 0.89 | 822 |
|  | Quartile 4 (n = 31) | 1.75 | (0.96, 3.28) | 0.07 | 822 |

*Note: All adjusted models controlled for IRC (country), maternal age at baseline, nulliparity, mother’s education, BMI at baseline, hemoglobin level at baseline, household food insecurity, mother’s diet diversity and whether there was a smoker presented at home.*

**Table S6.** Crude associations between weighted mean PM_2.5_/BC/CO exposures and fetal/neonatal outcomes.

| **Exposures** | **Model Type** | **Odds Ratio** | **95% CI** | **p-value** | **AIC** |
| --- | --- | --- | --- | --- | --- |
| *Congenital Abnormalities* | |  |  |  |  |
| PM_2.5_ | Linear | 1.00 | (1.00, 1.00) | 0.06 | 403 |
|  | Log linear | 1.36 | (0.89, 2.06) | 0.15 | 404 |
|  | Categorical [Ref. Quartile 1] (n = 10) |  |  |  |  |
|  | Quartile 2 (n = 6) | 0.59 | (0.20, 1.60) | 0.31 | 408 |
|  | Quartile 3 (n = 10) | 0.99 | (0.40, 2.43) | 0.98 | 408 |
|  | Quartile 4 (n = 12) | 1.19 | (0.51, 2.84) | 0.68 | 408 |
| BC | Linear | 1.02 | (0.98, 1.05) | 0.25 | 383 |
|  | Log linear | 1.27 | (0.78, 2.10) | 0.34 | 384 |
|  | Categorical [Ref. Quartile 1] (n = 9) |  |  |  |  |
|  | Quartile 2 (n = 11) | 1.21 | (0.50, 3.03) | 0.67 | 388 |
|  | Quartile 3 (n = 7) | 0.78 | (0.28, 2.10) | 0.62 | 388 |
|  | Quartile 4 (n = 9) | 0.99 | (0.38, 2.55) | 0.98 | 388 |
| CO | Linear | 0.97 | (0.83, 1.07) | 0.69 | 425 |
|  | Log linear | 1.10 | (0.84, 1.45) | 0.50 | 424 |
|  | Categorical [Ref. Quartile 1] (n = 8) |  |  |  |  |
|  | Quartile 2 (n = 16) | 1.97 | (0.86, 4.89) | 0.12 | 423 |
|  | Quartile 3 (n = 6) | 0.73 | (0.24, 2.11) | 0.56 | 423 |
|  | Quartile 4 (n = 10) | 1.23 | (0.48, 3.24) | 0.66 | 423 |
| *Stillbirth* |  |  |  |  |  |
| PM_2.5_ | Linear | 1.00 | (1.00, 1.00) | 0.42 | 298 |
|  | Log linear | 1.20 | (0.73, 1.99) | 0.47 | 298 |
|  | Categorical [Ref. Quartile 1] (n = 3) |  |  |  |  |
|  | Quartile 2 (n = 10) | 3.33 | (1.01, 14.9) | 0.07 | 298 |
|  | Quartile 3 (n = 5) | 1.66 | (0.41, 8.10) | 0.49 | 298 |
|  | Quartile 4 (n = 8) | 2.66 | (0.77, 12.2) | 0.15 | 298 |
| BC | Linear | 1.03 | (0.99, 1.06) | 0.08 | 284 |
|  | Log linear | 1.57 | (0.87, 2.89) | 0.14 | 284 |
|  | Categorical [Ref. Quartile 1] (n = 2) |  |  |  |  |
|  | Quartile 2 (n = 10) | 5.02 | (1.32, 32.7) | 0.04 | 285 |
|  | Quartile 3 (n = 7) | 3.54 | (0.85, 23.8) | 0.12 | 285 |
|  | Quartile 4 (n = 6) | 2.99 | (0.69, 20.5) | 0.18 | 285 |
| CO | Linear | 1.06 | (0.96, 1.12) | 0.13 | 317 |
|  | Log linear | 1.34 | (0.96, 1.89) | 0.09 | 316 |
|  | Categorical [Ref. Quartile 1] (n = 7) |  |  |  |  |
|  | Quartile 2 (n = 3) | 0.42 | (0.09, 1.50) | 0.20 | 316 |
|  | Quartile 3 (n = 6) | 0.83 | (0.27, 2.52) | 0.75 | 316 |
|  | Quartile 4 (n = 12) | 1.69 | (0.68, 4.58) | 0.27 | 316 |
| *Neonatal Death* | |  |  |  |  |
| PM_2.5_ | Linear | 1.00 | (1.00, 1.00) | 0.55 | 405 |
|  | Log linear | 1.36 | (0.89, 2.06) | 0.15 | 403 |
|  | Categorical [Ref. Quartile 1] (n = 7) |  |  |  |  |
|  | Quartile 2 (n = 7) | 1.00 | (0.34, 2.93) | 1.00 | 407 |
|  | Quartile 3 (n = 11) | 1.57 | (0.62, 4.29) | 0.35 | 407 |
|  | Quartile 4 (n = 13) | 1.87 | (0.76, 5.00) | 0.18 | 407 |
| BC | Linear | 1.01 | (0.97, 1.05) | 0.51 | 392 |
|  | Log linear | 1.30 | (0.80, 2.13) | 0.29 | 391 |
|  | Categorical [Ref. Quartile 1] (n = 7) |  |  |  |  |
|  | Quartile 2 (n = 9) | 1.29 | (0.48, 3.64) | 0.61 | 394 |
|  | Quartile 3 (n = 8) | 1.16 | (0.41, 3.32) | 0.78 | 394 |
|  | Quartile 4 (n = 13) | 1.87 | (0.76, 5.00) | 0.19 | 394 |
| CO | Linear | 0.99 | (0.85, 1.08) | 0.89 | 390 |
|  | Log linear | 1.18 | (0.89, 1.60) | 0.26 | 388 |
|  | Categorical [Ref. Quartile 1] (n = 5) |  |  |  |  |
|  | Quartile 2 (n = 12) | 2.35 | (0.87, 7.41) | 0.11 | 388 |
|  | Quartile 3 (n = 6) | 1.17 | (0.35, 4.07) | 0.80 | 388 |
|  | Quartile 4 (n = 13) | 2.60 | (0.97, 8.14) | 0.07 | 388 |
| *Composite Outcomes* | |  |  |  |  |
| PM_2.5_ | Linear | 1.00 | (1.00, 1.00) | 0.07 | 814 |
|  | Log linear | 1.34 | (1.02, 1.75) | 0.03 | 812 |
|  | Categorical [Ref. Quartile 1] (n = 18) |  |  |  |  |
|  | Quartile 2 (n = 21) | 1.18 | (0.62, 2.25) | 0.62 | 817 |
|  | Quartile 3 (n = 24) | 1.35 | (0.73, 2.55) | 0.34 | 817 |
|  | Quartile 4 (n = 29) | 1.63 | (0.91, 3.02) | 0.11 | 817 |
| BC | Linear | 1.02 | (0.99, 1.04) | 0.18 | 777 |
|  | Log linear | 1.32 | (0.97, 1.82) | 0.08 | 775 |
|  | Categorical [Ref. Quartile 1] (n = 17) |  |  |  |  |
|  | Quartile 2 (n = 27) | 1.62 | (0.88, 3.06) | 0.12 | 779 |
|  | Quartile 3 (n = 19) | 1.13 | (0.58, 2.21) | 0.72 | 779 |
|  | Quartile 4 (n = 25) | 1.49 | (0.80, 2.83) | 0.21 | 779 |
| CO | Linear | 1.00 | (0.93, 1.06) | 0.90 | 827 |
|  | Log linear | 1.16 | (0.97, 1.40) | 0.11 | 824 |
|  | Categorical [Ref. Quartile 1] (n = 17) |  |  |  |  |
|  | Quartile 2 (n = 29) | 1.72 | (0.95, 3.23) | 0.08 | 823 |
|  | Quartile 3 (n = 16) | 0.94 | (0.47, 1.87) | 0.85 | 823 |
|  | Quartile 4 (n = 31) | 1.84 | (1.02, 3.43) | 0.05 | 823 |

**Table S7.** Adjusted associations between weighted mean PM_2.5_/BC/CO exposures and fetal/neonatal outcomes in Guatemala

| **Exposures** | **Model Type** | **Odds Ratio** | **95% CI** | **p-value** | **AIC** |
| --- | --- | --- | --- | --- | --- |
| *Congenital Abnormalities* | |  |  |  |  |
| PM_2.5_ | Linear | 1.00 | (1.00, 1.00) | 0.52 | 184 |
|  | Log linear | 1.08 | (0.51, 2.28) | 0.84 | 185 |
| BC | Linear | 0.99 | (0.88, 1.06) | 0.82 | 177 |
|  | Log linear | 0.93 | (0.29, 2.87) | 0.90 | 177 |
| CO | Linear | 1.01 | (0.71, 1.27) | 0.95 | 186 |
|  | Log linear | 1.10 | (0.67, 1.87) | 0.71 | 185 |
| *Stillbirth* |  |  |  |  |  |
| PM_2.5_ | Linear | 1.00 | (0.98, 1.01) | 0.94 | 65 |
|  | Log linear | 1.03 | (0.16, 6.39) | 0.98 | 65 |
| BC | Linear | 0.91 | (0.62, 1.20) | 0.55 | 64 |
|  | Log linear | 0.67 | (0.04, 13.4) | 0.78 | 64 |
| CO | Linear | 1.13 | (0.49, 1.79) | 0.69 | 65 |
|  | Log linear | 1.31 | (0.39, 5.16) | 0.68 | 65 |
| *Neonatal Death* | |  |  |  |  |
| PM_2.5_ | Linear | 1.00 | (1.00, 1.00) | 0.38 | 129 |
|  | Log linear | 1.78 | (0.67, 4.96) | 0.26 | 128 |
| BC | Linear | 1.02 | (0.92, 1.07) | 0.64 | 128 |
|  | Log linear | 2.00 | (0.49, 7.46) | 0.32 | 128 |
| CO | Linear | 1.16 | (0.86, 1.43) | 0.22 | 129 |
|  | Log linear | 1.93 | (0.95, 4.12) | 0.08 | 127 |
| *Composite Outcomes* | |  |  |  |  |
| PM_2.5_ | Linear | 1.00 | (1.00, 1.00) | 0.48 | 253 |
|  | Log linear | 1.20 | (0.67, 2.18) | 0.54 | 253 |
| BC | Linear | 0.99 | (0.91, 1.05) | 0.85 | 245 |
|  | Log linear | 1.07 | (0.43, 2.61) | 0.88 | 245 |
| CO | Linear | 1.07 | (0.84, 1.26) | 0.52 | 254 |
|  | Log linear | 1.33 | (0.88, 2.06) | 0.19 | 253 |

*Note: We only present linear and log linear model results for IRC-specific adjusted associations due to small number of cases.*

*All adjusted models controlled for maternal age at baseline, nulliparity, mother’s education, BMI at baseline, hemoglobin level at baseline, household food insecurity, mother’s diet diversity and whether there was a smoker presented at home.*

**Table S8.** Adjusted associations between weighted mean PM_2.5_/BC/CO exposures and fetal/neonatal outcomes in India

| **Exposures** | **Model Type** | **Odds Ratio** | **95% CI** | **p-value** | **AIC** |
| --- | --- | --- | --- | --- | --- |
| *Congenital Abnormalities* | |  |  |  |  |
| PM_2.5_ | Linear | 0.99 | (1.00, 1.00) | 0.38 | 103 |
|  | Log linear | 0.70 | (0.25, 1.80) | 0.48 | 104 |
| BC | Linear | 0.99 | (0.89, 1.05) | 0.74 | 104 |
|  | Log linear | 0.83 | (0.32, 2.16) | 0.70 | 104 |
| CO | Linear | 0.85 | (0.40, 1.18) | 0.57 | 104 |
|  | Log linear | 1.03 | (0.64, 1.92) | 0.92 | 104 |
| *Stillbirth* |  |  |  |  |  |
| PM_2.5_ | Linear | 1.00 | (1.00, 1.00) | 0.08 | 101 |
|  | Log linear | 2.36 | (0.81, 6.81) | 0.11 | 101 |
| BC | Linear | 1.04 | (0.96, 1.11) | 0.20 | 102 |
|  | Log linear | 2.45 | (0.74, 8.93) | 0.16 | 101 |
| CO | Linear | 1.11 | (0.81, 1.32) | 0.37 | 104 |
|  | Log linear | 1.29 | (0.74, 2.43) | 0.39 | 103 |
| *Neonatal Death* | |  |  |  |  |
| PM_2.5_ | Linear | 1.00 | (0.98, 1.01) | 0.75 | 94 |
|  | Log linear | 1.22 | (0.40, 3.56) | 0.71 | 94 |
| BC | Linear | 1.03 | (0.95, 1.10) | 0.34 | 93 |
|  | Log linear | 1.72 | (0.59, 5.32) | 0.33 | 93 |
| CO | Linear | 0.92 | (0.44, 1.26) | 0.75 | 94 |
|  | Log linear | 1.20 | (0.68, 2.43) | 0.57 | 94 |
| *Composite Outcomes* | |  |  |  |  |
| PM_2.5_ | Linear | 1.00 | (1.00, 1.00) | 0.83 | 208 |
|  | Log linear | 1.19 | (0.63, 2.19) | 0.59 | 207 |
| BC | Linear | 1.02 | (0.96, 1.06) | 0.50 | 206 |
|  | Log linear | 1.28 | (0.68, 2.48) | 0.45 | 206 |
| CO | Linear | 1.00 | (0.75, 1.17) | 0.97 | 210 |
|  | Log linear | 1.13 | (0.81, 1.65) | 0.49 | 209 |

*Note: We only present linear and log linear model results for IRC-specific adjusted associations due to small number of cases.*

*All adjusted models controlled for maternal age at baseline, nulliparity, mother’s education, BMI at baseline, hemoglobin level at baseline, household food insecurity, mother’s diet diversity and whether there was a smoker presented at home.*

**Table S9.** Adjusted associations between weighted mean PM_2.5_/BC/CO exposures and fetal/neonatal outcomes in Peru

| **Exposures** | **Model Type** | **Estimate** | **95% CI** | **p-value** | **AIC** |
| --- | --- | --- | --- | --- | --- |
| *Congenital Abnormalities* | |  |  |  |  |
| PM_2.5_ | Linear | 1.01 | (1.00, 1.02) | 0.01 | 75 |
|  | Log linear | 1.48 | (0.54, 4.00) | 0.43 | 80 |
| BC | Linear | 1.04 | (0.91, 1.13) | 0.46 | 73 |
|  | Log linear | 1.03 | (0.32, 3.18) | 0.96 | 74 |
| CO | Linear | 0.99 | (0.75, 1.13) | 0.92 | 103 |
|  | Log linear | 1.12 | (0.61, 2.21) | 0.74 | 103 |
| *Stillbirth* |  |  |  |  |  |
| PM_2.5_ | Linear | 1.00 | (0.98, 1.01) | 0.86 | 85 |
|  | Log linear | 0.97 | (0.30, 2.76) | 0.96 | 85 |
| BC | Linear | 1.00 | (0.83, 1.11) | 0.96 | 75 |
|  | Log linear | 1.11 | (0.35, 3.54) | 0.85 | 75 |
| CO | Linear | 1.07 | (0.94, 1.15) | 0.15 | 91 |
|  | Log linear | 1.78 | (0.82, 4.06) | 0.16 | 90 |
| *Neonatal Death* | |  |  |  |  |
| PM_2.5_ | Linear | 1.00 | (0.98, 1.01) | 0.74 | 94 |
|  | Log linear | 0.94 | (0.35, 2.22) | 0.90 | 94 |
| BC | Linear | 0.93 | (0.74, 1.05) | 0.38 | 92 |
|  | Log linear | 0.69 | (0.24, 1.73) | 0.44 | 93 |
| CO | Linear | 0.99 | (0.73, 1.11) | 0.95 | 79 |
|  | Log linear | 1.27 | (0.62, 2.85) | 0.55 | 78 |
| *Composite Outcomes* | |  |  |  |  |
| PM_2.5_ | Linear | 1.00 | (1.00, 1.00) | 0.11 | 159 |
|  | Log linear | 1.26 | (0.69, 2.21) | 0.44 | 161 |
| BC | Linear | 1.00 | (0.92, 1.06) | 0.98 | 147 |
|  | Log linear | 0.95 | (0.49, 1.80) | 0.89 | 147 |
| CO | Linear | 1.03 | (0.92, 1.11) | 0.43 | 176 |
|  | Log linear | 1.31 | (0.86, 2.09) | 0.23 | 175 |

*Note: We only present linear and log linear model results for IRC-specific adjusted associations due to small number of cases.*

*All adjusted models controlled for maternal age at baseline, nulliparity, mother’s education, BMI at baseline, hemoglobin level at baseline, household food insecurity, mother’s diet diversity and whether there was a smoker presented at home.*

**Table S10.** Adjusted associations between weighted mean PM_2.5_/BC/CO exposures and fetal/neonatal outcomes in Rwanda

| **Exposures** | **Model Type** | **Estimate** | **95% CI** | **p-value** | **AIC** |
| --- | --- | --- | --- | --- | --- |
| *Congenital Abnormalities* | |  |  |  |  |
| PM_2.5_ | Linear | 1.00 | (0.99, 1.01) | 0.25 | 77 |
|  | Log linear | 3.58 | (0.82, 15.2) | 0.08 | 75 |
| BC | Linear | 1.10 | (1.00, 1.21) | 0.03 | 73 |
|  | Log linear | 4.56 | (0.65, 34.1) | 0.12 | 75 |
| CO | Linear | 0.98 | (0.49, 1.27) | 0.93 | 71 |
|  | Log linear | 1.27 | (0.49, 3.30) | 0.62 | 71 |
| *Stillbirth* |  |  |  |  |  |
| PM_2.5_ | Linear | 1.00 | (0.99, 1.01) | 0.44 | 100 |
|  | Log linear | 1.35 | (0.35, 5.07) | 0.66 | 101 |
| BC | Linear | 1.06 | (0.99, 1.13) | 0.04 | 96 |
|  | Log linear | 2.47 | (0.57, 9.75) | 0.21 | 98 |
| CO | Linear | 0.99 | (0.65, 1.21) | 0.95 | 110 |
|  | Log linear | 1.29 | (0.67, 2.53) | 0.46 | 110 |
| *Neonatal Death* | |  |  |  |  |
| PM_2.5_ | Linear | 1.00 | (0.99, 1.01) | 0.26 | 128 |
|  | Log linear | 2.38 | (0.79, 7.29) | 0.13 | 127 |
| BC | Linear | 1.03 | (0.92, 1.09) | 0.53 | 117 |
|  | Log linear | 2.28 | (0.63, 7.68) | 0.19 | 115 |
| CO | Linear | 0.76 | (0.42, 1.08) | 0.26 | 126 |
|  | Log linear | 0.79 | (0.47, 1.38) | 0.40 | 127 |
| *Composite Outcomes* | |  |  |  |  |
| PM_2.5_ | Linear | 1.00 | (1.00, 1.00) | 0.28 | 214 |
|  | Log linear | 1.90 | (0.89, 4.02) | 0.10 | 212 |
| BC | Linear | 1.04 | (0.98, 1.09) | 0.11 | 200 |
|  | Log linear | 2.02 | (0.84, 4.73) | 0.11 | 200 |
| CO | Linear | 0.92 | (0.69, 1.10) | 0.49 | 216 |
|  | Log linear | 1.04 | (0.70, 1.56) | 0.83 | 216 |

*Note: We only present linear and log linear model results for IRC-specific adjusted associations due to small number of cases.*

*All adjusted models controlled for maternal age at baseline, nulliparity, mother’s education, BMI at baseline, hemoglobin level at baseline, household food insecurity, mother’s diet diversity and whether there was a smoker presented at home.*

**Table S11**. Adjusted associations between mean post-intervention PM_2.5_/BC/CO exposures and fetal/neonatal outcomes.

| **Exposures** | **Model Type** | **Odds Ratio** | **95% CI** | **p-value** | **AIC** |
| --- | --- | --- | --- | --- | --- |
| *Congenital Abnormalities* | |  |  |  |  |
| PM_2.5_ | Linear | 1.00 | (1.00, 1.00) | 0.31 | 438 |
|  | Log linear | 1.12 | (0.78, 1.58) | 0.52 | 438 |
|  | Categorical [Ref. Quartile 1] |  |  |  |  |
|  | Quartile 2 | 1.07 | (0.40, 2.88) | 0.90 | 441 |
|  | Quartile 3 | 1.28 | (0.51, 3.37) | 0.60 | 441 |
|  | Quartile 4 | 1.52 | (0.62, 3.92) | 0.37 | 441 |
| BC | Linear | 1.02 | (0.99, 1.04) | 0.16 | 436 |
|  | Log linear | 1.06 | (0.72, 1.55) | 0.75 | 437 |
|  | Categorical [Ref. Quartile 1] |  |  |  |  |
|  | Quartile 2 | 0.90 | (0.34, 2.40) | 0.84 | 440 |
|  | Quartile 3 | 1.51 | (0.65, 3.69) | 0.34 | 440 |
|  | Quartile 4 | 1.06 | (0.42, 2.72) | 0.91 | 440 |
| CO | Linear | 0.94 | (0.75, 1.07) | 0.51 | 421 |
|  | Log linear | 1.14 | (0.94, 1.41) | 0.21 | 420 |
|  | Categorical [Ref. Quartile 1] |  |  |  |  |
|  | Quartile 2 | 1.46 | (0.48, 4.89) | 0.51 | 416 |
|  | Quartile 3 | 3.63 | (1.42, 11.1) | 0.01 | 416 |
|  | Quartile 4 | 1.42 | (0.47, 4.77) | 0.54 | 416 |
| *Stillbirth* |  |  |  |  |  |
| PM_2.5_ | Linear | 1.00 | (1.00, 1.00) | 0.32 | 406 |
|  | Log linear | 1.05 | (0.70, 1.55) | 0.80 | 406 |
|  | Categorical [Ref. Quartile 1] |  |  |  |  |
|  | Quartile 2 | 1.20 | (0.48, 3.06) | 0.70 | 410 |
|  | Quartile 3 | 0.98 | (0.36, 2.61) | 0.97 | 410 |
|  | Quartile 4 | 1.13 | (0.43, 2.96) | 0.81 | 410 |
| BC | Linear | 1.01 | (0.97, 1.04) | 0.41 | 405 |
|  | Log linear | 1.15 | (0.76, 1.71) | 0.50 | 405 |
|  | Categorical [Ref. Quartile 1] |  |  |  |  |
|  | Quartile 2 | 0.87 | (0.28, 2.65) | 0.80 | 401 |
|  | Quartile 3 | 2.56 | (1.08, 6.74) | 0.04 | 401 |
|  | Quartile 4 | 0.94 | (0.30, 2.88) | 0.91 | 401 |
| CO | Linear | 1.06 | (0.97, 1.12) | 0.11 | 429 |
|  | Log linear | 1.07 | (0.89, 1.30) | 0.51 | 430 |
|  | Categorical [Ref. Quartile 1] |  |  |  |  |
|  | Quartile 2 | 0.74 | (0.28, 1.89) | 0.53 | 433 |
|  | Quartile 3 | 0.74 | (0.28, 1.90) | 0.53 | 433 |
|  | Quartile 4 | 1.12 | (0.48, 2.68) | 0.80 | 433 |
| *Neonatal Death* | |  |  |  |  |
| PM_2.5_ | Linear | 1.00 | (1.00, 1.00) | 0.61 | 372 |
|  | Log linear | 1.24 | (0.82, 1.82) | 0.29 | 371 |
|  | Categorical [Ref. Quartile 1] |  |  |  |  |
|  | Quartile 2 | 2.37 | (0.86, 7.58) | 0.11 | 369 |
|  | Quartile 3 | 0.79 | (0.19, 3.04) | 0.73 | 369 |
|  | Quartile 4 | 2.45 | (0.88, 7.91) | 0.10 | 369 |
| BC | Linear | 1.00 | (0.95, 1.03) | 0.98 | 364 |
|  | Log linear | 0.98 | (0.62, 1.52) | 0.93 | 364 |
|  | Categorical [Ref. Quartile 1] |  |  |  |  |
|  | Quartile 2 | 1.52 | (0.54, 4.59) | 0.43 | 367 |
|  | Quartile 3 | 1.49 | (0.53, 4.50) | 0.46 | 367 |
|  | Quartile 4 | 1.33 | (0.45, 4.11) | 0.61 | 367 |
| CO | Linear | 0.98 | (0.81, 1.09) | 0.83 | 363 |
|  | Log linear | 1.01 | (0.83, 1.25) | 0.93 | 364 |
|  | Categorical [Ref. Quartile 1] |  |  |  |  |
|  | Quartile 2 | 0.68 | (0.22, 1.98) | 0.48 | 366 |
|  | Quartile 3 | 1.24 | (0.48, 3.30) | 0.65 | 366 |
|  | Quartile 4 | 0.96 | (0.35, 2.65) | 0.94 | 366 |
| *Composite Outcomes* | |  |  |  |  |
| PM_2.5_ | Linear | 1.00 | (1.00, 1.00) | 0.25 | 881 |
|  | Log linear | 1.11 | (0.88, 1.40) | 0.37 | 881 |
|  | Categorical [Ref. Quartile 1] |  |  |  |  |
|  | Quartile 2 | 1.52 | (0.85, 2.75) | 0.16 | 881 |
|  | Quartile 3 | 0.95 | (0.49, 1.81) | 0.87 | 881 |
|  | Quartile 4 | 1.52 | (0.85, 2.78) | 0.16 | 881 |
| BC | Linear | 1.00 | (0.98, 1.02) | 0.74 | 872 |
|  | Log linear | 1.03 | (0.80, 1.32) | 0.84 | 872 |
|  | Categorical [Ref. Quartile 1] |  |  |  |  |
|  | Quartile 2 | 1.25 | (0.67, 2.35) | 0.48 | 870 |
|  | Quartile 3 | 1.91 | (1.09, 3.45) | 0.03 | 870 |
|  | Quartile 4 | 1.11 | (0.59, 2.12) | 0.74 | 870 |
| CO | Linear | 1.01 | (0.93, 1.07) | 0.73 | 875 |
|  | Log linear | 1.06 | (0.94, 1.20) | 0.39 | 874 |
|  | Categorical [Ref. Quartile 1] |  |  |  |  |
|  | Quartile 2 | 0.87 | (0.47, 1.61) | 0.66 | 876 |
|  | Quartile 3 | 1.36 | (0.78, 2.41) | 0.28 | 876 |
|  | Quartile 4 | 1.00 | (0.55, 1.83) | 0.99 | 876 |

*Note: All adjusted models controlled for IRC (country), maternal age at baseline, nulliparity, mother’s education, BMI at baseline, hemoglobin level at baseline, household food insecurity, mother’s diet diversity and whether there was a smoker presented at home.*

**Table S12**. Assessment of effect modification by maternal age, baseline BMI and baseline gestational age

| **Interactions** | **Exposures** | **Model Type** | **Estimate** | **SE** | **p value** |
| --- | --- | --- | --- | --- | --- |
| *Congenital Abnormalities* | |  |  |  |  |
| age_med | PM | loglinear | -0.05 | 0.44 | 0.91 |
| age_med | BC | loglinear | -0.11 | 0.57 | 0.84 |
| age_med | CO | loglinear | 0.05 | 0.30 | 0.86 |
| ga_med | PM | loglinear | 0.32 | 0.43 | 0.46 |
| ga_med | BC | loglinear | 0.10 | 0.55 | 0.85 |
| ga_med | CO | loglinear | 0.04 | 0.30 | 0.89 |
| bmi_med | PM | loglinear | 0.58 | 0.43 | 0.18 |
| bmi_med | BC | loglinear | 0.17 | 0.55 | 0.76 |
| bmi_med | CO | loglinear | 0.19 | 0.30 | 0.53 |
| *Stillbirth* |  |  |  |  |  |
| age_med | PM | loglinear | 0.17 | 0.52 | 0.74 |
| age_med | BC | loglinear | -0.17 | 0.62 | 0.78 |
| age_med | CO | loglinear | 0.15 | 0.35 | 0.67 |
| ga_med | PM | loglinear | -0.31 | 0.52 | 0.56 |
| ga_med | BC | loglinear | -0.39 | 0.61 | 0.52 |
| ga_med | CO | loglinear | 0.40 | 0.35 | 0.25 |
| bmi_med | PM | loglinear | -0.72 | 0.54 | 0.18 |
| bmi_med | BC | loglinear | -1.05 | 0.62 | 0.09 |
| bmi_med | CO | loglinear | 0.43 | 0.35 | 0.22 |
| *Neonatal Death* | |  |  |  |  |
| age_med | PM | loglinear | 0.46 | 0.45 | 0.31 |
| age_med | BC | loglinear | 0.01 | 0.53 | 0.98 |
| age_med | CO | loglinear | -0.35 | 0.32 | 0.27 |
| ga_med | PM | loglinear | 0.16 | 0.44 | 0.72 |
| ga_med | BC | loglinear | -0.10 | 0.52 | 0.85 |
| ga_med | CO | loglinear | 0.02 | 0.34 | 0.95 |
| bmi_med | PM | loglinear | 0.52 | 0.45 | 0.25 |
| bmi_med | BC | loglinear | 0.36 | 0.54 | 0.51 |
| bmi_med | CO | loglinear | 0.03 | 0.33 | 0.93 |
| *Composite Outcomes* | |  |  |  |  |
| age_med | PM | loglinear | 0.12 | 0.29 | 0.67 |
| age_med | BC | loglinear | -0.18 | 0.35 | 0.61 |
| age_med | CO | loglinear | -0.04 | 0.20 | 0.84 |
| ga_med | PM | loglinear | 0.19 | 0.28 | 0.49 |
| ga_med | BC | loglinear | 0.06 | 0.34 | 0.86 |
| ga_med | CO | loglinear | 0.08 | 0.20 | 0.70 |
| bmi_med | PM | loglinear | 0.31 | 0.28 | 0.27 |
| bmi_med | BC | loglinear | 0.03 | 0.35 | 0.92 |
| bmi_med | CO | loglinear | 0.19 | 0.20 | 0.33 |

*Note: Values in estimate indicate the coefficients of the interaction term between assessed potential effect modifier and weighted average exposure to PM_2.5_/BC/CO. Effect modification assessed using log linear exposure models and controlled for IRC (country), maternal age at baseline, nulliparity, mother’s education, BMI at baseline, hemoglobin level at baseline, household food insecurity, mother’s diet diversity and whether there was a smoker presented at home.*


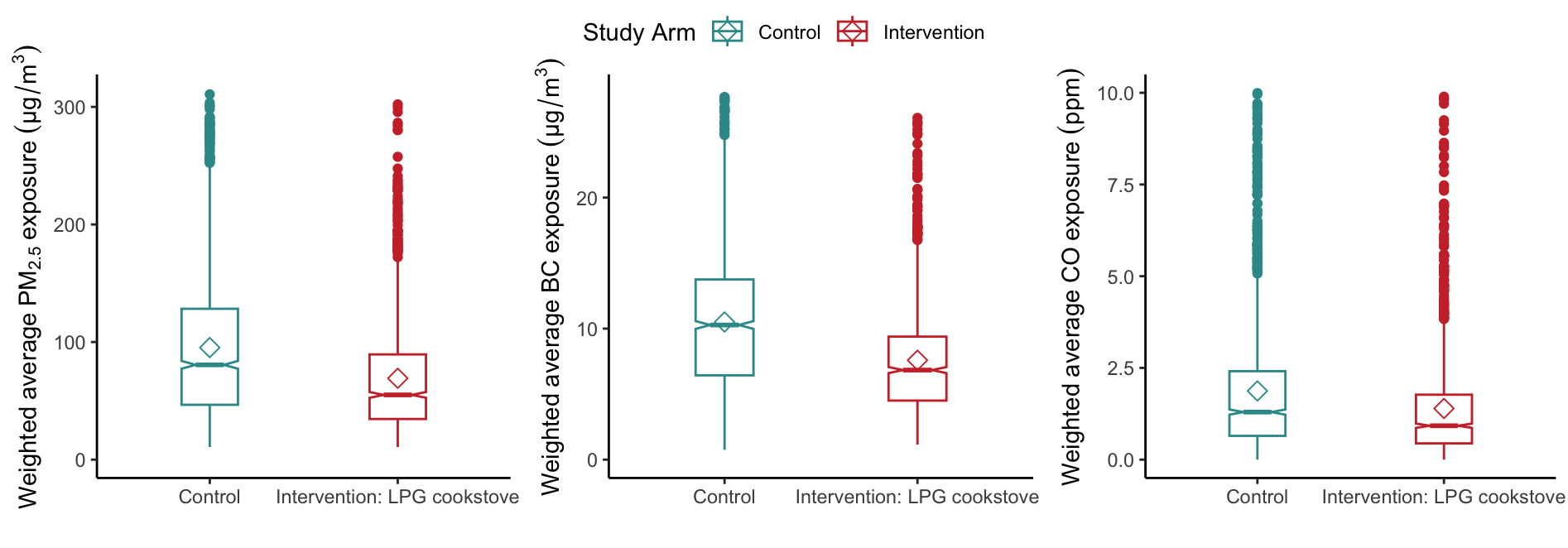


**Figure S1**. Box plots of weighted mean PM_2.5_/BC/CO exposures over pregnancy by arm. The square in each box indicates the mean value.
